# Novel Imidazopyridine–Oxadiazole β‑Tubulin Inhibitors Suppress Breast Cancer Migration and Induce Caspase-3-Mediated Apoptosis

**DOI:** 10.1021/acsomega.5c10113

**Published:** 2026-01-02

**Authors:** Mustafa Cakir, Burak Kuzu

**Affiliations:** † Department of Medical Biology, Faculty of Medicine, 668505 Van Yuzuncu Yil University, Van 65080, Türkiye; ‡ Department of Pharmaceutical Chemistry, Faculty of Pharmacy, 53000 Van Yuzuncu Yil University, Van 65080, Türkiye

## Abstract

A series of novel imidazo­[1,2-*a*]­pyridine–oxadiazole (**iMPZ1–15**) hybrid compounds was designed as potential β-tubulin polymerization inhibitors, inspired by the cis-conformation and biaryl scaffold of combretastatin A-4. The effects of **iMPZ** beta-tubulin inhibitors on proliferation in MDA-MB-231, SH-SY5Y, and DLD-1 cancer cells, as well as their influence on beta-tubulin inhibition, colonization, cell migration, cell cycle progression, and apoptosis in MDA-MB-231 cells, were investigated. **iMPZ-8** identified as the most efficacious treatment candidate, with an IC_50_ value of 7.5 μM in MDA MB-231 cells. **iMPZ-8** had a comparable effectiveness to NOC, which served as a positive control for beta tubulin inhibition. **IMPZ-8** reduced cellular migration and colonization. It also accumulated throughout the G2/M phase of the cell cycle, through the BAX-Caspase-3 intrinsic apoptotic signaling pathway.

## Graphical abstract

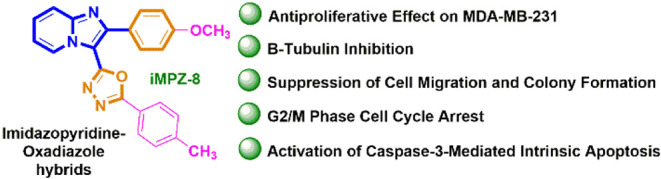

## Introduction

Microtubules are critical cytoskeletal elements that play a vital role in maintaining structural integrity and facilitating numerous essential biological processes in eukaryotic cells.^1^ These include cell formation, division, shape maintenance, intracellular vesicle transport, and cellular motility.^2^ Additionally, microtubules are involved in regulating programmed cell death (apoptosis) through intracellular signaling pathways.^3^ The dynamic behavior of microtubules—driven by cycles of polymerization and depolymerization—is essential for the proper organization of mitotic spindles and chromosomal segregation.^4^ Disruption of this dynamic balance leads to the arrest of cell division at the metaphase stage by impairing chromosome alignment and spindle formation, ultimately resulting in mitotic blockade and cell death.^5^ This mechanism has become a key focus in the development of novel, targeted anticancer therapies.^6^

Tubulin, the fundamental structural unit of microtubules, has three major ligand-binding domains: the taxane, vinca, and colchicine binding sites.^7^ Agents that target the taxane site act as microtubule-stabilizing agents by preventing depolymerization, while compounds binding to the vinca or colchicine sites inhibit tubulin polymerization and consequently arrest cells in mitosis.^8^ Among natural compounds that bind to the colchicine site are colchicine, steganacin, podophyllotoxin, and combretastatins, all of which share common structural features such as a biaryl scaffold^9^ (Figure 1). Within this group, Combretastatin A-4 (CA-4) stands out for its potent antitubulin activity. CA-4 binds at the interface of the α,β-tubulin heterodimer, inhibiting the conformational flexibility of β-tubulin and thereby preventing the formation of the straight heterodimer conformation required for microtubule assembly.^10^

**Figure 1:**
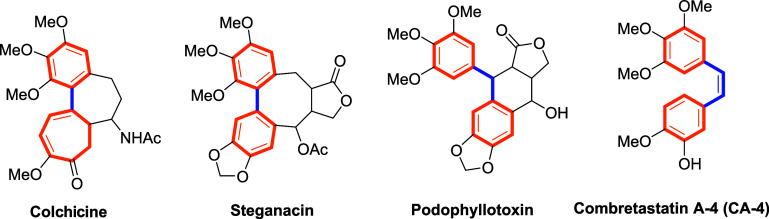
Naturally occurring tubulin polymerization inhibitors.

However, **CA-4** and similar natural antitubulin agents are limited by poor aqueous solubility, low bioavailability, and short biological half-life, all of which restrict their therapeutic utility.^11^ To overcome these pharmacokinetic drawbacks, several synthetic derivatives have been developed in recent years based on the core structural features of these natural compounds. Among them, **CA-4**-inspired analogs—such as nocodazole and compound **1**—have demonstrated potent inhibition of microtubule assembly through tubulin binding and show strong therapeutic potential across various cancer types.^12,13^ These compounds typically preserve the cis-configuration of the biaryl core found in CA-4 through linkers such as alkenes, aryls, or heteroaryl groups (Figure 2).

**Figure 2:**
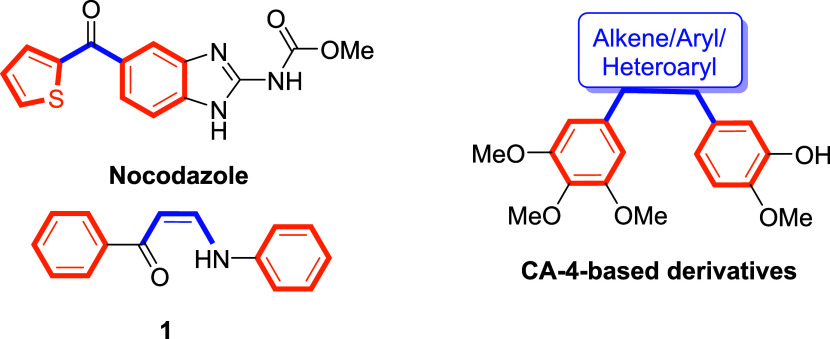
Synthetic compounds developed as tubulin polymerization inhibitors.

Among synthetic **CA-4** derivatives, those utilizing an imidazopyridine ring to maintain the essential cis-configuration have recently garnered attention.^14^ In these structures, the phenyl group at the 2-position of the imidazopyridine core is retained, while the 3-position is functionalized with various substituents such as guanylhydrazones (**2**), anilines (**3**), propenones (**4**), oxindoles (**5**), or benzimidazoles (**6**).^15–17^ These modifications have been reported to impart strong antitubulin activity and promising anticancer properties. Additionally, imidazopyridine–oxadiazole hybrids (**7**), which adopt a more linear geometry akin to nocodazole, have also shown significant antitubulin effects.^18^ Notably, hybrid structures combining oxadiazole rings with imidazopyridine isosteres (**8**) have demonstrated meaningful levels of antiproliferation^19^ (Figure 3).

**Figure 3:**
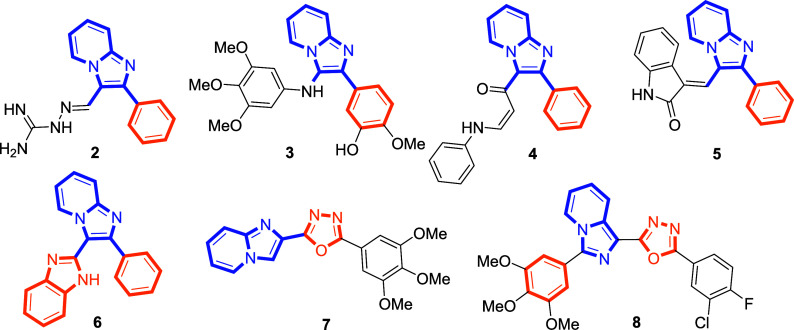
Representative structures of imidazopyridine-based agents.

Building on these findings, the current study aims to explore the antitubulin potential of novel compounds incorporating an imidazopyridine ring as the structural motif to enforce cis-conformation within biaryl systems. We designed a series of derivatives by retaining a phenyl ring at the 2-position of the imidazopyridine scaffold and introducing various modifications. Inspired particularly by reports highlighting the synergistic potential of oxadiazole-imidazopyridine hybrids, we designed—for the first time—a novel series of compounds with oxadiazole units introduced at the 3-position of the imidazopyridine ring (Figure 4). This rational design was employed to evaluate their potential as tubulin polymerization inhibitors, with the overarching goal of developing novel antitubulin agents for anticancer therapy.

**Figure 4:**
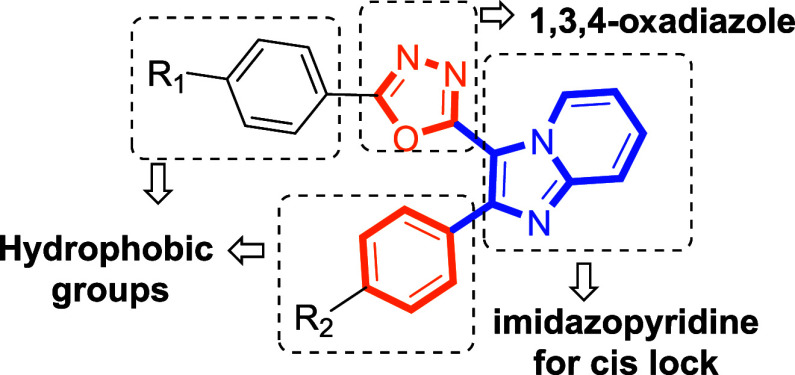
Rational design of novel imidazopyridine–oxadiazole hybrid compounds.

## Results and Discussions

### Chemistry

The synthetic route employed to obtain the target compounds is illustrated in Figure 5 and comprises four sequential steps. Initially, aryl-substituted imidazopyridine derivatives (**11a**–**e**) were synthesized in high yield via the reaction of 2-aminopyridine (**9**) with α-haloketones (**10a**–**e**). In the second step, these imidazopyridine intermediates (**11a**–**e**) were converted into aldehyde-substituted imidazopyridines (**12a**–**e**) through a Vilsmeier–Haack reaction using DMF and POCl_3_. Subsequently, various para-substituted benzohydrazide derivatives (**13a**–**e**) underwent condensation with the aldehyde-substituted imidazopyridines (**12a**–**e**) in ethanol under acetic acid catalysis. Finally, the resulting intermediates (**14a**–**o**) were cyclized into 1,3,4-oxadiazole derivatives (**iMPZ1–15**) through treatment with molecular iodine in a basic medium using potassium carbonate (K_2_CO_3_) as the base (Figure 5). The chemical structures of all synthesized compounds were rigorously elucidated and unequivocally confirmed by extensive spectroscopic and spectrometric analyses, including ^1^H NMR, ^13^C NMR, and high-resolution mass spectrometry (HRMS).

**Figure 5:**
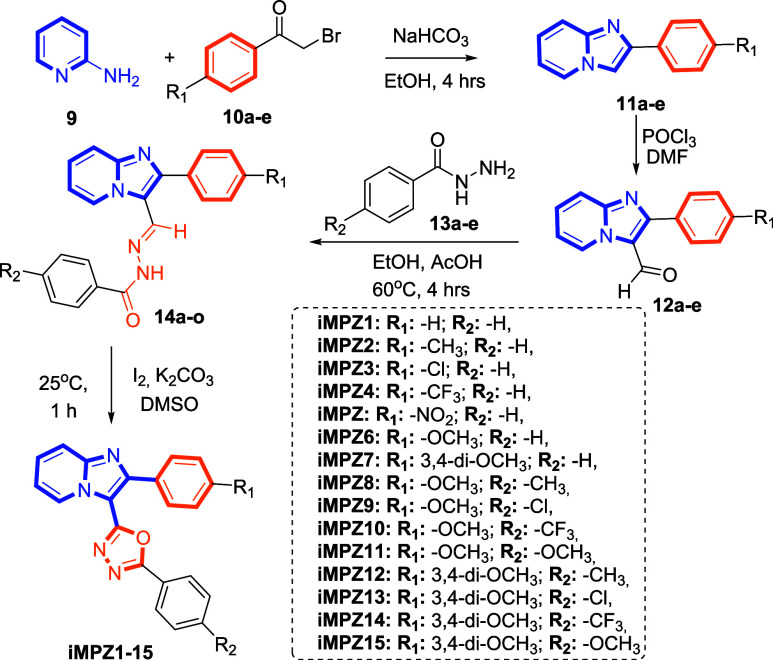
General synthesis scheme of imidazopyridine–oxadiazole hybrid compounds.

### Assessment of Cancer Cell Proliferation Inhibition

The effects of IMPZ compounds, at concentrations ranging from 1 to 80 μM, were assessed on SH-SY5Y, MDA-MB-231, and DLD-1 cell lines over a period of 72 h. **iMPZ-8** exhibited the lowest IC_50_ concentration in SH-SY5Y and MDA-MB-231 cells, and **iMPZ-1** demonstrated the lowest IC_50_ value in DLD-1 cells (Table 1). **iMPZ-8** and **iMPZ-3** were identified as the most stable and effective compounds among the three distinct cancer cell lines. The 50% inhibitory concentrations at 72 h were found to be 7.5, 9.2, and 19.7 μM for **iMPZ-8** and 9.7, 12.8, and 21.4 μM for **iMPZ-3** in MDA-MB-231, SH-SY5Y, and DLD-1 cells, respectively (Figure 6a,b). ***iMPZ-8*** displayed the strongest inhibitory effect on MDA-MB-231 breast cancer cells and showed minimal toxicity to healthy epithelial breast cells (Figure 6). The selectivity index (SI) for this compound was approximately 7.

**Table 1:** Human Breast Cancer (MDA MB 231), Human Neuroblastoma (SH-SY5Y), and Human Colon Cancer (DLD-1) Cell Lines Were Administered Treatment with **IMPZ-1–15** for a Total of 72 H, Followed by Analysis Using the MTT Assay to Assess Cell Viability^a^

	IC_50_ (μM)		
Compounds	MDA-MB-231	SH-SY5Y	DLD-1
**iMPZ-1**	**12.5**	**10.2**	**14.3**
**iMPZ-2**	26.7	21.8	47.3
**iMPZ-3**	**9.7**	**12.8**	**21.4**
**iMPZ-4**	38.6	45.1	67.5
**iMPZ-5**	56.4	58.9	100<
**iMPZ-6**	26.4	28.9	40.3
**iMPZ-7**	34.2	57.1	78.7
**iMPZ-8**	**7**.**5**	**9.2**	**19.7**
**iMPZ-9**	22.5	18.9	36.6
**iMPZ-10**	32.2	24.5	36.9
**iMPZ-11**	26.5	22.8	100<
**iMPZ-12**	**14.8**	**10.3**	**19.6**
**iMPZ-13**	55.3	72.4	100<
**iMPZ-14**	58.6	66.1	100<
**iMPZ-15**	26.5	22.8	87.6

a IC_50_ values were determined for each cell type. Groups exhibiting IC_50_ values below 20 μM are highlighted in bold red , and subsequent studies were conducted with these compounds.

**Figure 6:**
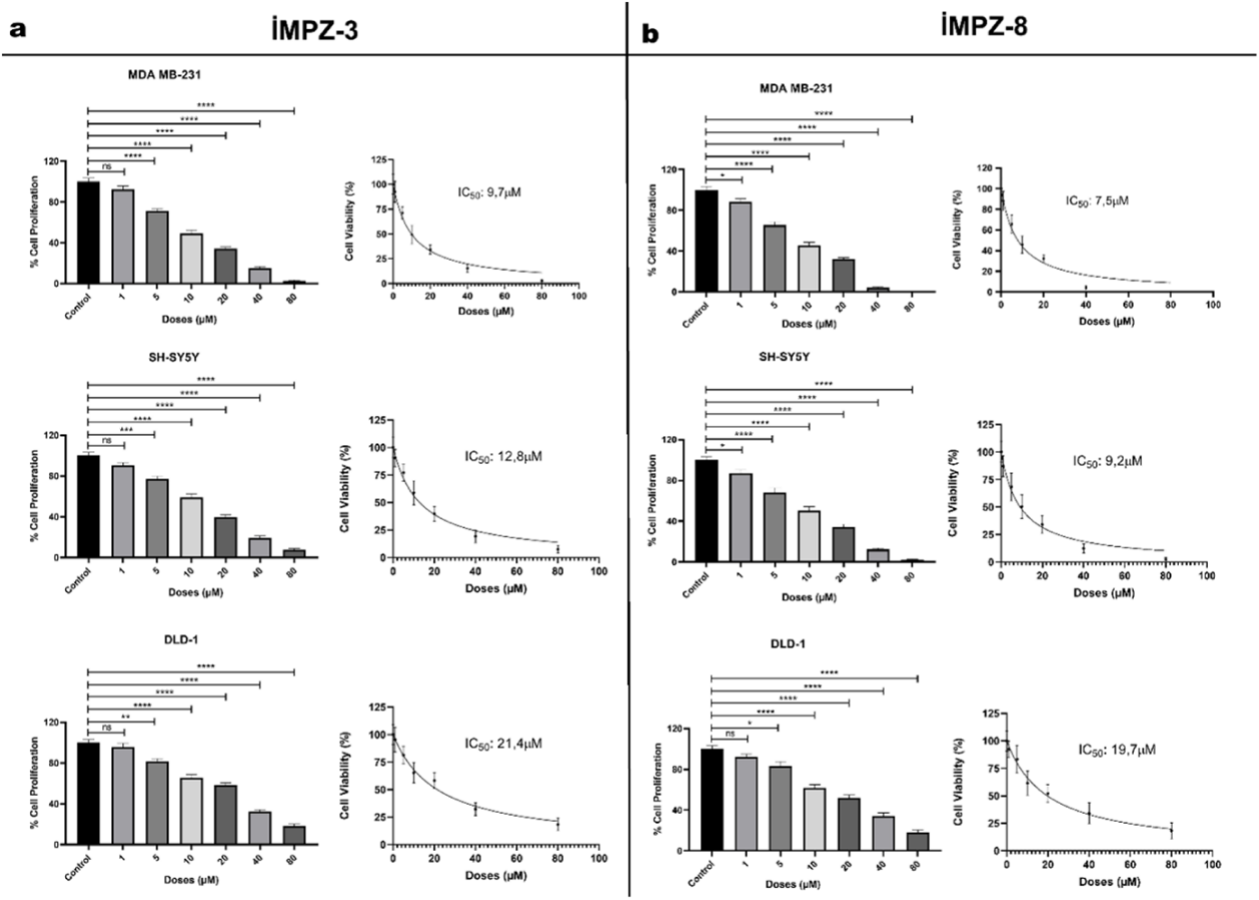
Impact of **iMPZ-3** and **iMPZ-8** molecules on cell proliferation in breast cancer, neuroblastoma, and colon cancer is examined. (a) The effects of **iMPZ-3** within the 1–80 μM concentration range on cell proliferation throughout three distinct cancer cell lines in a 72 h period is given. (b) The effects of IMPZ-8 on cell proliferation in three distinct cancer cell lines within the 1–80 μM dose range over a 72-h period are given. The dose–response curves and IC_50_ values were assessed for the two compounds following 72 h. *n* = 3, three replicates, *: *p* ≤ 0.05, **: *p* ≤ 0.01, ***: *p* ≤ 0.001, ****: *p* ≤ 0.0001.

The antiproliferative activities of the synthesized **iMPZ** compounds reveal clear structure–activity relationships. **iMPZ-8**, bearing a methoxy group at R1 and a methyl group at R2, exhibited the most potent activity across all tested cancer cell lines, suggesting that these electron-donating substituents enhance efficacy. Similarly, compounds with a methoxy group at R1, such as **iMPZ-12**, showed relatively strong activity, highlighting the favorable effect of methoxy substitution at this position. In contrast, compounds containing multiple methoxy substituents (e.g., **iMPZ-13**, **iMPZ-14**, **iMPZ-15**) demonstrated reduced antiproliferative effects, possibly due to steric hindrance or excessive electron density. Electron-withdrawing groups such as nitro (**iMPZ-5**) and trifluoromethyl (**iMPZ-4**) generally correlated with lower activity. Moreover, the presence of small substituents at both R1 and R2 positions (as in **iMPZ-1** and **iMPZ-3**) was associated with better activity compared to bulkier or multiple substitutions. Overall, these results suggest that the combination of electron-donating groups at R1, particularly methoxy, along with small substituents at R2, optimizes the antiproliferative potency of the **iMPZ** derivatives.

### Verifying the Effectiveness of Potential Beta-Tubulin Inhibitors in Breast Cancer Cells

Nocodazole (NOC), a β-tubulin antagonist, served as a positive control. MDA MB 231, SH-SY5Y, and DLD-1 cells were subjected to treatment with 3 μM NOC and compounds **iMPZ-1**, **iMPZ-3**, and **iMPZ-8** for 48 h, following which β-tubulin levels were quantified from cell lysates via a β-tubulin-specific ELISA technique. Compounds **iMPZ-1**, **iMPZ-3**, **iMPZ-8,** and NOC markedly inhibited β-tubulin protein expression relative to the untreated control group (Figure 7a). Notably, **iMPZ-8** exhibited the most significant inhibitory effects on β-tubulin expression and microtubule organization, comparable to those observed for Nocodazole (NOC), a well-known β-tubulin antagonist used here as a positive control. To further investigate this effect, immunofluorescence staining utilizing a β-tubulin-specific antibody was conducted, and the mean fluorescence indices were evaluated (Figure 7b). Numerous antimitotic agents, including nocodazole, are documented in the literature, targeting tubulin and functioning by altering microtubule organization.^20^ To evaluate the impact of **iMPZ-8** and NOC on microtubule architecture and distribution, cells were stained with β-tubulin (FITC-green) and nuclei (DAPI-blue) and examined using a fluorescence microscope. ImageJ was utilized for analysis to ascertain the distribution of β-tubulin on a per-cell basis and its expression levels within the cell. Cells treated with **iMPZ-8** and NOC exhibited a further reduction in β-tubulin levels and a notable disruption of microtubule structure (Figure 7b). **iMPZ-8** demonstrates antiproliferative activity comparable to established medicinal products such as NOC and alters microtubule structure, indicating its potential as a powerful antimitotic agent.

**Figure 7:**
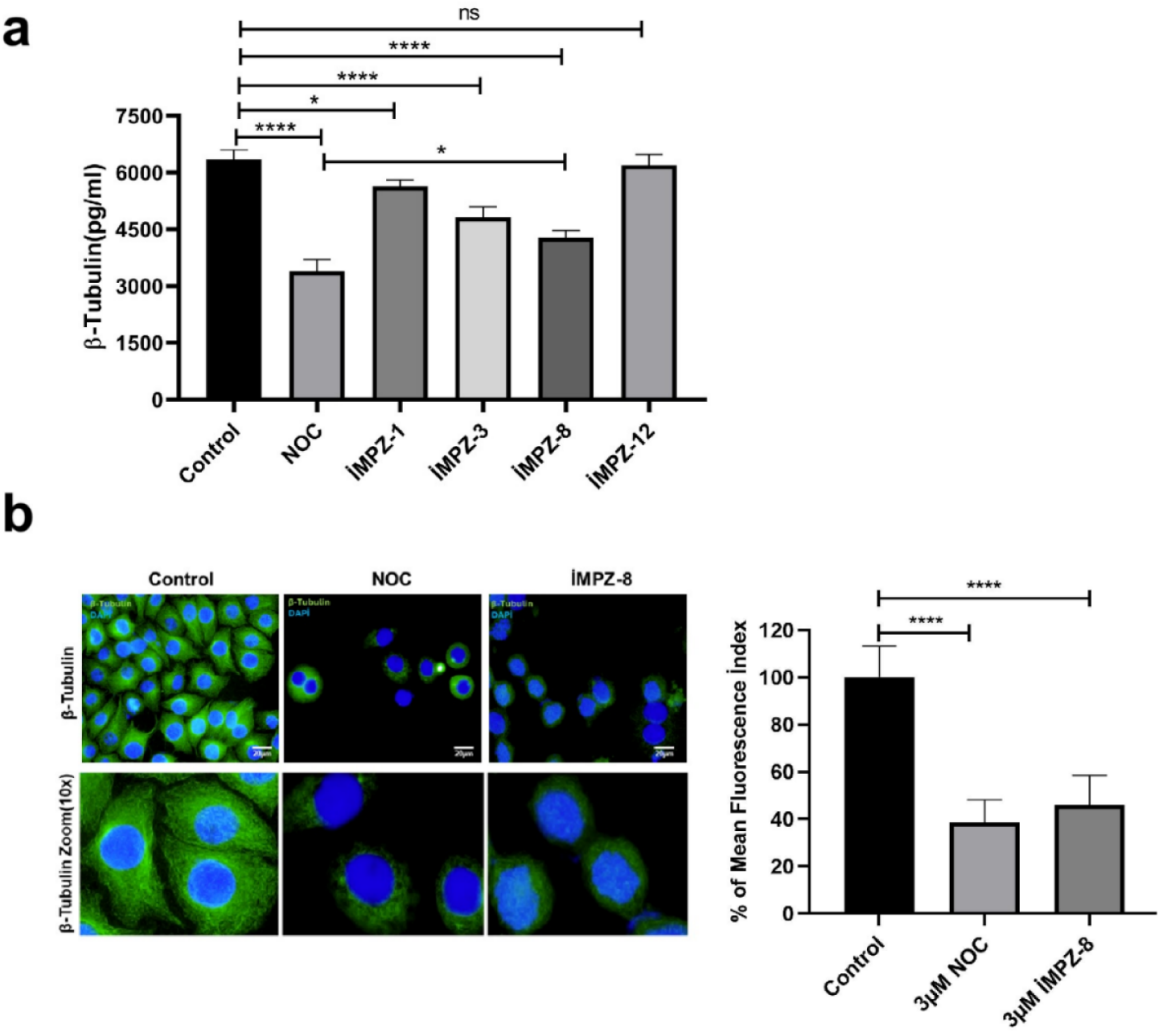
Distribution of β-tubulin protein expression inside cells. (A) The expression levels of β-tubulin protein in MDA MB-231 cells were assessed after the administration of 3 μM NOC and **iMPZ-1**, **iMPZ-3**, **iMPZ-8**, and **iMPZ-12** molecules. (B) Cells exhibited immunofluorescence staining following treatment with NOC (3 μM) and **iMPZ-8** (3 μM). Areas of β-tubulin coupled with FITC are depicted in green. Regions stained with DAPI denote cell nuclei (Scale bar: 20 μm). NOC: Nocadazole, *n* = 3, three replicates, *: *p* ≤ 0.05, **: *p* ≤ 0.01, ***: *p* ≤ 0.001, ****: *p* ≤ 0.0001.

### The β-Tubulin Inhibitor iMPZ-8 Induces Intrinsic Apoptosis in Breast Cancer Cells by Obstructing Migration and Colonization, While Modulating the Cell Cycle

Microtubules, hollow filaments composed of α-tubulin and β-tubulin heterodimers, are vital constituents of the cytoskeleton.^21^ Microtubules are essential for controlling cell migration, promoting cell adhesion, delivering new membrane components, and supporting sustained cell extensions during wound healing.^22^ The impact of the **iMPZ-8** molecule’s attenuation of β-tubulin on cellular migration and colonization in cancer cells was examined. To observe cell migration, cells were seeded, then a wound was created, and the wound was observed for 48 h. The wound diameter greatly decreased in the compounds-free group, whereas it remained somewhat wide in the NOC and **iMPZ-8** groups (Figure 8a). **iMPZ-8** decreased cell migration by almost 50% relative to the control group. A similar effect on cell migration was likewise seen in the colonization of neoplastic cells. **iMPZ-8** reduced cell collagenity by nearly 30% relative to untreated cancer cells (Figure 8b). The prevention of colonization and migration of MDA MB-231 cells by **iMPZ-8** is attributed to its suppressive action on β-tubulin.

**Figure 8:**
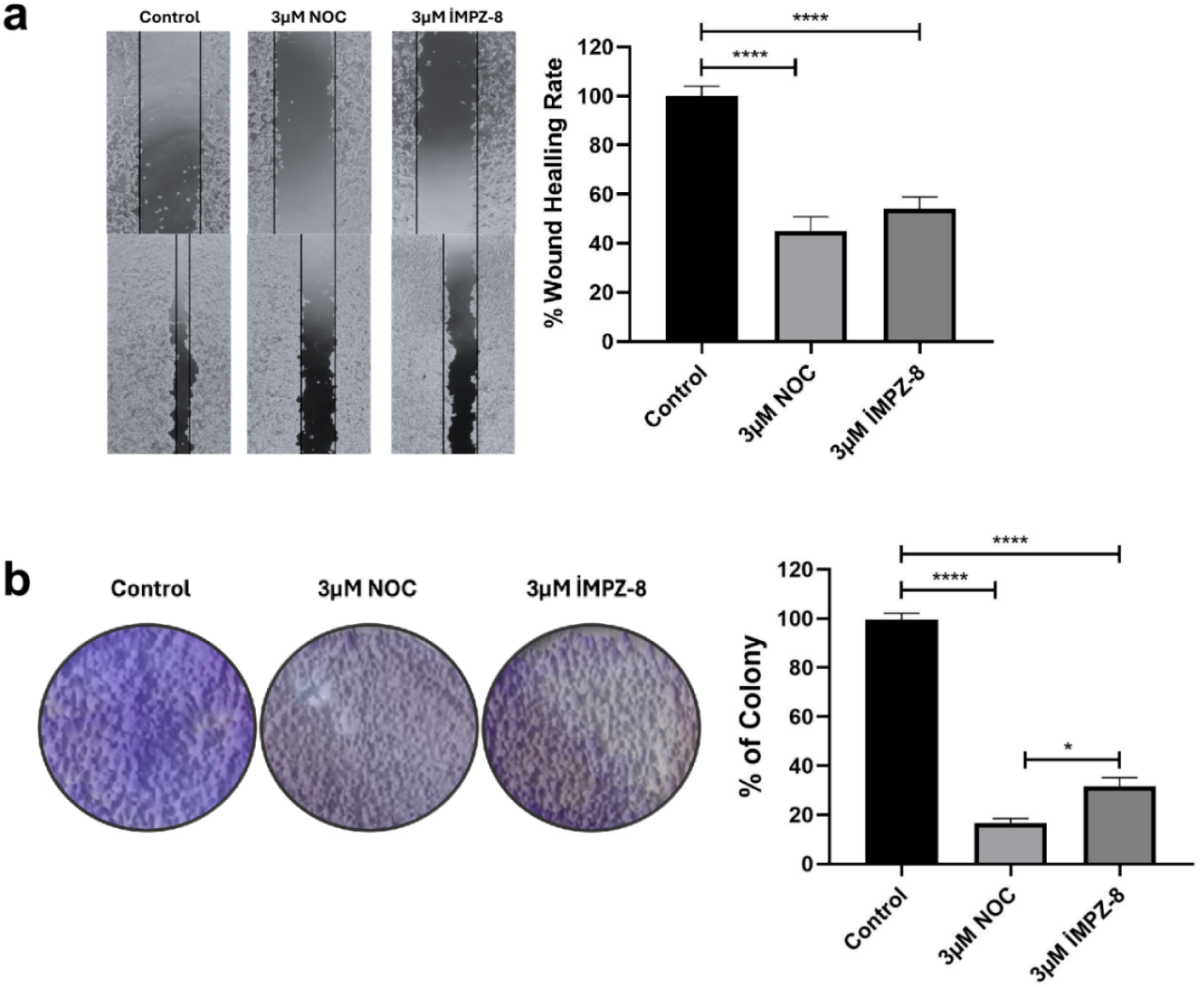
Demonstrating the impact of β-tubulin inhibitors on the migration and colonization of MDA MB-231 cancer cells. (A) Illustrating movement in breast cancer cells, (B) Assessing the impact on the colony-forming capabilities of breast cancer cells. NOC: Nocadazole, *n* = 3, three replicates, *: *p* ≤ 0.05, **: *p* ≤ 0.01, ****: *p* ≤ 0.0001.

Remarkably evolved α- and β-tubulin heterodimers combine into functional microtubules, executing various critical cellular functions, including structural support and force generation during cell division.^2^ The application of nocodazole, an instance of microtubule inhibitor, triggers the accumulation of cancer cells in the G2/M phase.^23^ Cancer cells were stained with PI and investigated by flow cytometry^24^ to determine the stage of the cell cycle at which the B tubulin inhibitor caused cell retention. The cells exhibit behavior analogous to NOC, resulting in increased accumulation during the G2/M phase (Figure 9a). Cancer comprises cells that proliferate incessantly and excessively; hence, there are cell cycle checkpoints and DNA surveillance mechanisms that inhibit the accumulation and dissemination of genetic mistakes during cell division. These checkpoints can impede cell cycle progression or induce cell cycle exit or apoptosis in response to catastrophic DNA damage.^25^ To determine in the event cell death occurred via apoptosis as a result of the accumulation of breast cancer cells at different stages of the cell cycle, the cells were stained with PI and Annexin V and subsequently analyzed using flow cytometry.^26^ The flow cytometry analysis indicated that NOC and **iMPZ-8** cells exhibited accumulation in both early and late apoptosis as compared to the untreated group (Figure 9b). The expression levels of particular pro-apoptotic and antiapoptotic genes were analyzed to confirm that **iMPZ-8** undergoes apoptosis. The results complemented those obtained from flow cytometry, suggesting that the cells engaged the intrinsic apoptosis pathway through BAX-Caspase 3, resulting in the regulated death of cancer cells (Figure 9c).

**Figure 9:**
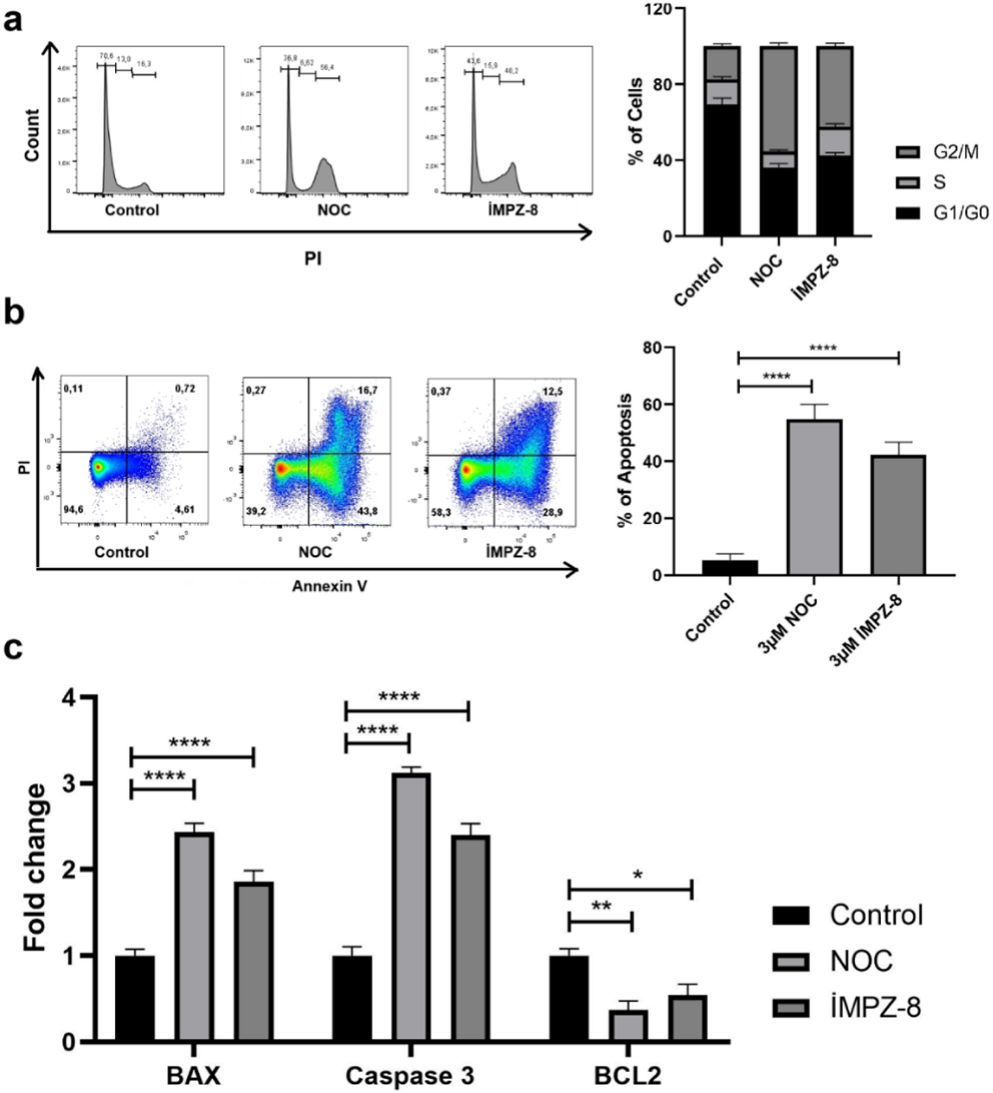
Microtubule inhibitor **iMPZ-8** induces intrinsic apoptosis in cells by arresting the cell cycle at the G2/M phase. (A) Analysis of cell cycle phases through PI staining of MDA MB-231 cells, (B) Assessment of MDA MB-231 cells stained with PI and Annexin V via flow cytometry to observe early and late apoptosis stages, (C) Evaluation of pro-apoptotic and antiapoptotic gene expression in MDA MB-231 cells, with normalization conducted using GAPDH. NOC: Nocadazole, *n* = 3, three replicates, *: *p* ≤ 0.05, **: *p* ≤ 0.01, ***: *p* ≤ 0.001, ****: *p* ≤ 0.0001.

## Conclusion

In this study, a novel series of imidazopyridine–oxadiazole derivatives were rationally designed and synthesized to act as tubulin polymerization inhibitors with potential antitumor properties. By retaining the phenyl group at the 2-position of the imidazopyridine scaffold and introducing oxadiazole moieties at the 3-position, a unique structural motif was developed to preserve the essential cis-conformation necessary for effective β-tubulin binding. Among the synthesized compounds, **iMPZ-8** emerged as the most promising candidate, exhibiting potent antiproliferative activity in MDA-MB-231, SH-SY5Y, and DLD-1 cancer cells, with a particularly low IC_50_ value (7.5 μM) in triple-negative breast cancer cells. Further in vitro investigations confirmed that **iMPZ-8** significantly inhibited β-tubulin expression, disrupted microtubule architecture, and demonstrated comparable efficacy to nocodazole. Functional assays revealed that **iMPZ-8** effectively suppressed cellular migration and colony formation, suggesting a strong impact on cancer cell invasiveness. Flow cytometry and immunofluorescence analyses indicated that **iMPZ-8** induced G2/M phase arrest and activated intrinsic apoptosis via the BAX–Caspase-3 signaling pathway. Importantly, **iMPZ-8** exhibited a high selectivity index, suggesting limited toxicity toward normal breast epithelial cells. Taken together, these findings suggest that the imidazopyridine–oxadiazole scaffold represents a promising chemotype for the development of novel microtubule-targeting agents. **iMPZ-8**, in particular, shows strong potential as a β-tubulin inhibitor with dual antimitotic and pro-apoptotic effects, meriting further investigation for preclinical development as a targeted anticancer therapeutic.

## Experimental Section

### Chemistry

#### Synthesis of Aryl-Substituted Imidazopyridine Derivatives (**11a–e**)

Phenacyl bromide derivatives (**10a**–**e**) and sodium bicarbonate (1.5 mmol) were added to the solution of 2-aminopyridine^9^ (1 mmol) in ethanol (10 mL). The reaction mixture was stirred at 65 °C for 4 h and then at room temperature for an additional 2 h. The completion of the reaction was monitored by TLC control, and after the reaction was terminated, the solvent was removed under a reduced vacuum. Twenty ml of water was added to the raw product and mixed for 30 min at room temperature and solid particles were formed. The precipitate was collected via filtration and dried for further use.^27^

#### Synthesis of Carbaldehyde-Substituted Imidazopyridine Derivatives (**12a–e**)

A total of 2 mmol of the previously obtained substituted imidazopyridine derivatives (**11a**–**e**) was dissolved in 4 mL of DMF and cooled to 0 °C in an ice bath. To generate the Vilsmeier–Haack reagent, phosphoryl chloride (POCl_3_) was added dropwise to the stirring solution. The reaction mixture was stirred at this temperature for 1 h, then heated to 80 °C under reflux and stirred overnight. The reaction progress was monitored by TLC. Upon completion, the reaction mixture was poured into 50 mL of an ice–water bath. The resulting precipitate was collected by filtration and air-dried at room temperature. The crude product was used in the subsequent step without further purification.^28^

#### Synthesis of Hydrazone-Linked Imidazopyridine Derivatives (**14a–o**)

A solution of aldehyde-substituted imidazopyridine derivatives (**12a–e**, 1 mmol) in 10 mL of ethanol was prepared, followed by the addition of 1 mmol of the corresponding benzohydrazide derivatives (**13a**–**e**) dissolved in 5 mL of ethanol, along with a catalytic amount of acetic acid. The reaction mixture was stirred at 60 °C for 4 h. TLC monitored progress. Upon completion, the reaction mixture was poured into 50 mL of an ice–water mixture, resulting in the formation of solid precipitates. The solids were collected by filtration and washed several times with a water–ethanol mixture. The crude products (**14a**–**o**) were air-dried and used in the next step without further purification.

#### Synthesis of Target Compounds (**iMPZ1–15**)

In the final step, compounds **14a–o** (1 mmol) were dissolved in 5 mL of DMSO. To this solution, potassium carbonate (K_2_CO_3_, 3 mmol) was added, and the mixture was stirred at room temperature for 5 min. Molecular iodine (1.2 mmol) was then added, and the reaction was refluxed for 6 h using a magnetic stirrer under a condenser. Reaction progress was monitored by thin-layer chromatography (TLC). Upon confirming complete conversion of the starting material, the reaction mixture was cooled to room temperature and poured into 100 mL of an ice–water mixture. The resulting precipitate was collected by filtration, washed thoroughly with water (3 × 10 mL), and air-dried. The crude product was further purified by silica gel column chromatography using an appropriate ethyl acetate/*n*-hexane solvent system.^29^

#### Characterization Data of Final Compounds

##### 2-Phenyl-5-(2-phenylimidazo­[1,2-*a*]­pyridin-3-yl)-1,3,4-oxadiazole (**iMPZ-1**)

Light gray solid, M.p.: 152.5–152.9 °C, Yield: 79%. Rf: 0.57 (ethyl acetate/*n*-hexane, 1/2). ^1^H NMR (400 MHz, CDCl_3_) δ 9.58 (dt, *J* = 1.1 Hz, *J* = 6.9 Hz, 1H, Ar–H), 7.84–7.73 (m, 5H, Ar–H), 7.50–7.37 (m, 7H, Ar–H), 7.08 (td, *J* = 1.1 Hz, *J* = 6.9 Hz, 1H, Ar–H). ^13^C NMR (100 MHz, CDCl_3_) δ 161.9, 156.9, 149.2, 146.3, 132.5, 130.8, 128.7, 128.2, 128.1, 127.4, 127.2, 126.8, 125.8, 122.4, 116.5, 113.4, 105.9. HRMS [M + H]; Calculated for C_21_H_15_N_4_O: 339.1240, Found: 339.1246.

##### 2-Phenyl-5-(2-(p-tolyl)­imidazo­[1,2-*a*]­pyridin-3-yl)-1,3,4-oxadiazole (**iMPZ-2**)

White solid, M.p.: 207.4–207.9 °C, Yield: 81%. Rf: 0.60 (ethyl acetate/*n*-hexane, 1/2). ^1^H NMR (400 MHz, CDCl_3_) δ 9.57 (dt, *J* = 1.2 Hz, *J* = 7.0 Hz, 1H, Ar–H), 7.86–7.78 (m, 2H, Ar–H), 7.76–7.67 (m, 3H, Ar–H), 7.47–7.37 (m, 4H, Ar–H), 7.31–7.24 (m, AA’BB’ system, 2H, Ar–H), 7.05 (td, *J* = 1.2 Hz, *J* = 7.0 Hz, 1H, Ar–H), 2.42 (s, 3H, −CH_3_). ^13^C NMR (100 MHz, CDCl_3_) δ 162.9, 158.1, 150.5, 147.3, 139.2, 131.7, 130.6, 129.6, 129.1, 128.9, 128.4, 127.7, 126.8, 123.5, 117.5, 114.2, 106.7, 21.5. HRMS [M + H]; Calculated for C_22_H_17_N_4_O: 353.1396, Found: 353.1402.

##### 2-(2-(4-Chlorophenyl)­imidazo­[1,2-*a*]­pyridin-3-yl)-5-phenyl-1,3,4-oxadiazole (**iMPZ-3**)

Yellow solid, M.p.: 219.1–219.6 °C, Yield: 88%. Rf: 0.55 (ethyl acetate/*n*-hexane, 1/2). ^1^H NMR (400 MHz, CDCl_3_) δ 9.63 (quasi dt, *J* = 6.9 Hz, 1H, Ar–H), 7.89–7.87 (m, AA’BB’ system, 2H, Ar–H), 7.85–7.78 (m, 3H, Ar–H), 7.55–7.47 (m, 6H, Ar–H), 7.15 (td, *J* = 1.1 Hz, *J* = 6.9 Hz, 1H, Ar–H). ^13^C NMR (100 MHz, CDCl_3_) δ 163.0, 157.8, 148.9, 147.3, 135.4, 132.1, 131.9, 131.0, 129.2, 128.5, 128.4, 128.0, 126.8, 123.3, 117.6, 114.5, 106.9. Calculated for C_21_H_14_ClN_4_O: 373.0851, Found: 373.0866.

##### 2-Phenyl-5-(2-(4-(trifluoromethyl)­phenyl)­imidazo­[1,2-a]­pyridin-3-yl)-1,3,4-oxadiazole (**iMPZ-4**)

White solid, M.p.: 217.3–217.8 °C, Yield: 72%. Rf: 0.62 (ethyl acetate/*n*-hexane, 1/2). ^1^H NMR (400 MHz, CDCl_3_) δ 9.55 (dt, *J* = 1.2 Hz, *J* = 6.9 Hz, 1H, Ar–H), 7.95–7.88 (m, AA’BB’ system, 2H, Ar–H), 7.79–7.68 (m, 5H, Ar–H), 7.47–7.43 (m, 2H, Ar–H), 7.41–7.37 (m, 2H, Ar–H), 7.11 (td, *J* = 1.2 Hz, *J* = 6.9 Hz, 1H, Ar–H). ^13^C NMR (100 MHz, CDCl_3_) δ 163.0, 157.5, 148.5, 147.4, 137.3, 131.9, 131.1 (d, J = 32.6), 130.1, 129.2, 128.4, 128.1, 126.7, 125.1 (q, *J* = 3.7 Hz), 123.2, 122.8, 117.8, 114.7, 107.4. Calculated for C_22_H_14_F_3_N_4_O: 407.1114, Found: 407.1121.

##### 2-(2-(4-Nitrophenyl)­imidazo­[1,2-*a*]­pyridin-3-yl)-5-phenyl-1,3,4-oxadiazole (**iMPZ-5**)

Yellow solid, M.p.: 238.1–238.5 °C, Yield: 63%. Rf: 0.45 (ethyl acetate/*n*-hexane, 1/2). ^1^H NMR (400 MHz, CDCl_3_) δ 10.04 (bs, 1H, Ar–H), 9.65–9.58 (m, 1H, Ar–H), 8.37–8.28 (m, 3H, Ar–H), 8.14–7.88 (m, 3H, Ar–H), 7.84–7.73 (m, 2H, Ar–H), 7.62–7.54 (m, 1H, Ar–H), 7.50–7.41 (m, 1H, Ar–H), 7.14 (td, *J* = 1.0 Hz, *J* = 6.9 Hz, 1H, Ar–H). ^13^C NMR (100 MHz, CDCl_3_) δ 178.7, 155.2, 148.6, 147.8, 138.7, 131.0, 130.6, 129.3, 128.9, 128.5, 126.7, 124.1, 123.4, 117.9, 117.8, 116.1, 115.0. HRMS [M + H]; Calculated for C_21_H_14_N_5_O_3_: 384.1091, Found: 384.1098.

##### 2-(2-(4-Methoxyphenyl)­imidazo­[1,2-*a*]­pyridin-3-yl)-5-phenyl-1,3,4-oxadiazole (**iMPZ-6**)

Light gray solid, M.p.: 174.6–174.9 °C, Yield: 77%. Rf: 0.37 (ethyl acetate/*n*-hexane, 1/2). ^1^H NMR (400 MHz, CDCl_3_) δ 9.56 (quasi dt, *J* = 6.9 Hz, 1H, Ar–H), 7.86–7.83 (m, 2H, Ar–H), 7.78–7.75 (m, AA’BB’ system, 2H, Ar–H), 7.72 (dt, *J* = 9.0 Hz, 1H, Ar–H), 7.47–7.38 (m, 4H, Ar–H), 7.05 (td, *J* = 1.1 Hz, *J* = 6.9 Hz, 1H, Ar–H). 7.02–6.98 (m, AA’BB’ system, 2H, Ar–H), 3.85 (s, 3H, -OCH_3_). ^13^C NMR (100 MHz, CDCl_3_) δ 162.8, 160.5, 158.1, 150.2, 147.3, 131.7, 131.1, 129.1, 128.4, 127.7, 126.8, 126.0, 123.5, 117.4, 114.1, 113.6, 106.4, 55.5. HRMS [M + H]; Calculated for C_22_H_17_N_4_O_2_: 369.1346, Found: 369.1352.

##### 2-(2-(3,4-Dimethoxyphenyl)­imidazo­[1,2-*a*]­pyridin-3-yl)-5-phenyl-1,3,4-oxadiazole (**iMPZ-7**)

Light brown solid, M.p. :184.5–185.1 °C, Yield: 71%. Rf: 0.25 (ethyl acetate/*n*-hexane, 1/2). ^1^H NMR (400 MHz, CDCl_3_) δ 9.54 (quasi dt, *J* = 0.9 Hz, *J* = 6.9 Hz, 1H, Ar–H), 7.83–7.81 (m, 2H, Ar–H), 7.71 (dd, *J* = 0.9 Hz, *J* = 9.0 Hz, 1H, Ar–H), 7.46–7.36 (m, 6H, Ar–H), 7.06–7.02 (m, 1H, Ar–H), 6.98–6.91 (m, 1H, Ar–H), 3.92 (s, 3H, -OCH_3_), 3.84 (s, 3H, -OCH_3_). ^13^C NMR (100 MHz, CDCl_3_) δ 162.8, 158.1, 150.2, 150.0, 148.7, 147.2, 131.8, 129.1, 128.3, 127.8, 126.7, 126.1, 123.4, 122.6, 117.4, 114.2, 112.5, 110.6, 106.4, 56.1, 56.0. HRMS [M + H]; Calculated for C_23_H_19_N_4_O_3_: 399.1451, Found: 399.1459.

##### 2-(2-(4-Methoxyphenyl)­imidazo­[1,2-*a*]­pyridin-3-yl)-5-(p-tolyl)-1,3,4-oxadiazole (**iMPZ-8**)

Yellow solid, M.p.: 199.4–200.0 °C, Yield: 76%. Rf: 0.43 (ethyl acetate/*n*-hexane, 1/2). ^1^H NMR (400 MHz, CDCl_3_) δ 9.55 (dt, *J* = 1.1 Hz, *J* = 6.9 Hz, 1H, Ar–H), 7.77–7.68 (m, 5H, Ar–H), 7.38 (ddd, *J* = 1.2 Hz, *J* = 6.9 Hz, *J* = 9.0 Hz, 1H, Ar–H), 7.20–7.18 (m, 2H, Ar–H), 7.03 (td, *J* = 1.2 Hz, *J* = 6.9 Hz, 1H, Ar–H), 7.00–6.96 (m, AA’BB’ system, 2H, Ar–H), 3.85 (s, 3H, -OCH_3_), 2.33 (s, 3H, −CH_3_). ^13^C NMR (100 MHz, CDCl_3_) δ 163.0, 160.5, 157.9, 150.0, 147.2, 142.3, 131.1, 129.8, 128.4, 127.6, 126.8, 126.0, 120.7, 117.3, 114.1, 113.6, 106.5, 55.5, 21.7. HRMS [M + H]; Calculated for C_23_H_19_N_4_O_2_: 383.1502, Found: 383.1509.

##### 2-(4-Chlorophenyl)-5-(2-(4-methoxyphenyl)­imidazo­[1,2-*a*]­pyridin-3-yl)-1,3,4-oxadiazole (**iMPZ-9**)

White solid, M.p.: 186.4–187.0 °C, Yield: 79%. Rf: 0.40 (ethyl acetate/*n*-hexane, 1/2). ^1^H NMR (400 MHz, CDCl_3_) δ 9.55 (dt, *J* = 1.1 Hz, *J* = 6.9 Hz, 1H, Ar–H), 7.78–7.71 (m, 5H, Ar–H), 7.43–7.37 (m, 3H, Ar–H), 7.38 (td, *J* = 1.1 Hz, *J* = 6.9 Hz, 1H, Ar–H), 7.01–6.97 (m, AA’BB’ system, 2H, Ar–H), 3.85 (s, 3H, -OCH_3_). ^13^C NMR (100 MHz, CDCl_3_) δ 161.0, 159.5, 157.2, 149.3, 146.3, 137.0, 130.0, 128.5, 127.3, 127.0, 126.8, 126.3, 124.9, 120.9, 116.4, 113.2, 112.6, 55.4. HRMS [M + H]; Calculated for C_22_H_16_ClN_4_O_2_: 403.0956, Found: 403.0963.

##### 2-(2-(4-Methoxyphenyl)­imidazo­[1,2-*a*]­pyridin-3-yl)-5-(4-(trifluoromethyl)­phenyl)-1,3,4-oxadiazole (**iMPZ-10**)

Light gray solid, M.p.: 187.8–188.2 °C, Yield: 69%. Rf: 0.45 (ethyl acetate/*n*-hexane, 1/2). ^1^H NMR (400 MHz, CDCl_3_) δ 9.56 (quasi dt, *J* = 0.9 Hz, *J* = 6.9 Hz, 1H, Ar–H), 7.95 (d, *J* = 8.1 Hz, 2H, Ar–H), 7.77–7.72 (m, 3H, Ar–H), 7.67 (d, *J* = 8.1 Hz, 2H, Ar–H), 7.42 (quasi td, *J* = 0.9 Hz, *J* = 6.9 Hz, 1H, Ar–H), 7.07 (td, *J* = 0.9 Hz, *J* = 6.9 Hz, 1H, Ar–H), 7.02–6.98 (m, AA’BB’ system, 2H, Ar–H), 3.86 (s, 3H, -OCH_3_). ^13^C NMR (100 MHz, CDCl_3_) δ 161.6, 160.7, 158.6, 150.7, 133.4, 133.1, 131.0, 128.4, 128.0, 127.1, 126.7, 126.2 (q, *J* = 3.7 Hz), 128.5, 124.9, 122.2, 117.5, 114.3, 113.6, 55.5. HRMS [M + H]; Calculated for C_23_H_16_F_3_N_4_O_2_: 437.1220, Found: 437.1225.

##### 2-(4-Methoxyphenyl)-5-(2-(4-methoxyphenyl)­imidazo­[1,2-*a*]­pyridin-3-yl)-1,3,4-oxadiazole (**iMPZ-11**)

Green solid, M.p.: 197.9–198.5 °C, Yield: 74%. Rf: 0.30 (ethyl acetate/*n*-hexane, 1/2). ^1^H NMR (400 MHz, CDCl_3_) δ 9.58 (m, 1H, Ar–H), 7.79–7.75 (m, 4H, Ar–H), 7.71–7.69 (m, 1H, Ar–H), 7.38 (t, *J* = 8.4 Hz, 1H, Ar–H), 7.05–6.98 (m, 3H, Ar–H), 6.89 (dd, *J* = 2.4 Hz, *J* = 8.8 Hz, 2H, Ar–H), 3.85 (s, 3H, -OCH_3_), 3.79 (s, 3H, -OCH_3_). ^13^C NMR (100 MHz, CDCl_3_) δ 162.8, 162.4, 160.5, 157.6, 149.8, 147.2, 131.1, 128.6, 128.3, 127.6, 126.0, 117.3, 116.0, 114.6, 114.1, 113.6, 106.5, 55.5, 55.4. HRMS [M + H]; Calculated for C_23_H_19_N_4_O_3_: 399.1451, Found: 399.1459.

##### 2-(2-(3,4-Dimethoxyphenyl)­imidazo­[1,2-*a*]­pyridin-3-yl)-5-(p-tolyl)-1,3,4-oxadiazole (**iMPZ-12**)

Light gray solid, M.p.:216.2–216.7 °C, Yield: 78%. Rf: 0.18 (ethyl acetate/*n*-hexane, 1/2). ^1^H NMR (400 MHz, CDCl_3_) δ 9.61 (quasi dt, *J* = 0.8 Hz, *J* = 6.9 Hz, 1H, Ar–H), 7.85 (m, 3H, Ar–H), 7.49–7.45 (m, 3H, Ar–H), 7.28–7.26 (m, 2H, Ar–H), 7.11 (quasi td, *J* = 1.0 Hz, *J* = 6.9 Hz, 1H, Ar–H), 7.03–7.01 (m, 1H, Ar–H), 4.01 (s, 3H, -OCH_3_), 3.92 (s, 3H, -OCH_3_), 2.41 (3H, −CH_3_). ^13^C NMR (100 MHz, CDCl_3_) δ 163.0, 157.8, 150.0, 149.9, 148.7, 147.1, 142.4, 129.8, 128.3, 127.7, 126.7, 126.1, 122.6, 120.6, 117.3, 114.2, 112.5, 110.6, 106.5, 56.1, 56.0, 21.7. HRMS [M + H]; Calculated for C_24_H_21_N_4_O_3_: 413.1608, Found: 413.1613.

##### 2-(4-Chlorophenyl)-5-(2-(3,4-dimethoxyphenyl)­imidazo­[1,2-*a*]­pyridin-3-yl)-1,3,4-oxadiazole (**iMPZ-13**)

Yellow solid, M.p.:229.7–230.2 °C, Yield: 83%. Rf: 0.20 (ethyl acetate/*n*-hexane, 1/2). ^1^H NMR (400 MHz, CDCl_3_) δ 9.54 (d, *J* = 6.8 Hz, 1H, Ar–H), 7.76–7.72 (m, 3H, Ar–H), 7.44–7.34 (m, 5H, Ar–H), 7.06 (t, *J* = 6.8 Hz, 1H, Ar–H), 6.94 (d, *J* = 8.6 Hz, 1H, Ar–H), 3.93 (s, 3H, -OCH_3_), 3.85 (s, 3H, -OCH_3_). ^13^C NMR (100 MHz, CDCl_3_) δ 161.0, 157.2, 149.4, 149.0, 147.7, 137.1, 128.5, 127.3, 126.9, 126.8, 125.0, 121.6, 120.8, 116.4, 113.3, 111.5, 109.5, 55.1, 55.0. HRMS [M + H]; Calculated for C_23_H_18_ClN_4_O_3_: 433.1061, Found: 433.1070.

##### 2-(2-(3,4-Dimethoxyphenyl)­imidazo­[1,2-*a*]­pyridin-3-yl)-5-(4-(trifluoromethyl)­phenyl)-1,3,4-oxadiazole (**iMPZ-14**)

White solid, M.p.:212.1–212.5 °C, Yield: 71%. Rf: 0.22 (ethyl acetate/*n*-hexane, 1/2). ^1^H NMR (400 MHz, CDCl_3_) δ 9.53 (dt, *J* = 1.0 Hz, *J* = 6.9 Hz, 1H, Ar–H), 7.94–7.92 (d, *J* = 8.2 Hz, 2H, Ar–H), 7.73 (dt, *J* = 1.0 Hz, *J* = 9.0 Hz, 1H, Ar–H), 7.66 (d, *J* = 8.2 Hz, 2H, Ar–H), 7.45–7.41 (m, 1H, Ar–H), 7.39–7.32 (m, 2H, Ar–H), 7.07 (td, *J* = 1.0 Hz, *J* = 6.9 Hz, 1H, Ar–H), 6.96–6.94 (m, 1H, Ar–H), 3.94 (s, 3H, -OCH_3_), 3.86 (s, 3H, -OCH_3_). ^13^C NMR (100 MHz, CDCl_3_) δ 161.6, 158.6, 150.7, 150.2, 148.8, 147.4, 133.5, 133.2, 128.3, 128.2, 127.0, 126.6, 126.2 (q, J= 3.6 Hz), 126.0, 122.6, 117.5, 114.4, 112.6, 110.6, 106.2, 56.2, 56.1. HRMS [M + H]; Calculated for C_24_H_18_F_3_N_4_O_3_: 467.1325, Found: 467.1330.

##### 2-(2-(3,4-Dimethoxyphenyl)­imidazo­[1,2-*a*]­pyridin-3-yl)-5-(4-methoxyphenyl)-1,3,4-oxadiazole (**iMPZ-15**)

Yellow solid, M.p.:199.8–200.3 °C, Yield: 74%. Rf: 0.15 (ethyl acetate/*n*-hexane, 1/2). ^1^H NMR (400 MHz, CDCl_3_) δ 9.53 (quasi dt, *J* = 6.9 Hz, 1H, Ar–H), 7.77–7.75 (m, AA’BB’ system, 2H, Ar–H), 7.71 (d, *J* = 9.0 Hz, 1H, Ar–H), 7.41–7.37 (m, 3H, Ar–H), 7.06–7.02 (m, 1H, Ar–H), 6.95 (d, *J* = 8.1 Hz, 1H, Ar–H), 6.90–6.88 (m, AA’BB’ system, 2H, Ar–H), 3.93 (s, 3H, -OCH_3_), 3.84 (s, 3H, -OCH_3_), 3.79 (s, 3H, -OCH_3_). ^13^C NMR (100 MHz, CDCl_3_) δ 162.8, 162.4, 157.6, 149.9, 149.8, 148.7, 147.1, 128.5, 128.3, 127.6, 126.2, 122.6, 117.3, 115.9, 114.6, 114.1, 112.6, 110.7, 106.5, 56.1, 56.0, 55.5. HRMS [M + H]; Calculated for C_24_H_21_N_4_O_4_: 429.1557, Found: 429.1563.

### Biology

#### Cell Culture and Cell Proliferation

Human neuroblastoma (SH-SY5Y), human breast cancer (MDA-MB-231), and human colon cancer (DLD-1) cell lines obtained from the American Type Culture Collection (ATCC, Manassas, VA) were utilized. SH-SY5Y, MDA-MB-231, and DLD-1 were cultured in Dulbecco’s Modified Eagle Medium (DMEM) supplemented with 10% fetal bovine serum (FBS), 4.5 g/L l-glutamine, sodium pyruvate, 100 units/ml penicillin/streptomycin, and 1% Amphotericin B in an incubator maintained at 37 °C and 5% CO_2_. The impact of synthesized **iMPZ** beta-tubulin inhibitors on cell growth was assessed using the MTT (3-(4,5-dimethylthiazol-2-yl)-2,5-diphenyltetrazolium bromide) test. All cells were inoculated at a density of 5 × 10^4^ cells per well in a 96-well culture plate with candidate compounds (**iMPZ-1–15**) at varying concentrations from 1 to 80 μM. A 5 mg/mL MTT solution was introduced to each well containing the cells, which were incubated for 72 h followed by an additional 4-h incubation. Subsequently, 100 μL of DMSO was introduced, and viability assessments were conducted by detecting absorbance at 490 nm **(21.28).**

#### Colony Formation Assay

A colony formation assay was conducted to evaluate the impact of **iMPZ-8** and nocodazole on the colonization of breast cancer cells. MDA MB-231 cells were seeded at a density of 1000 cells per six wells. After 48 h, once the cells had adhered and initiated colony formation, 3 μM **iMPZ-8** or nocodazole was administered. Following a duration of 7 to 10 days, the medium in the 6-well plate was removed. The specimen was subsequently preserved with methanol and stained using a combination of 2% crystal violet and methanol. After 5 min, the stain was eliminated and rinsed three times with PBS. The colonies were counted and assessed **(28).**

#### Wound Healling Assay

MDA MB-231 cells were inoculated in 6-well plates at a density of 10 × 10^5^ cells per well. After 48 h, when the cell density had attained around 70%, wounds were created using a 10 μL pipet tip. **iMPZ-8** and nocodazole were subsequently introduced. Images were captured at 0 and 48 h. The investigation was concluded when the control group exhibited significant closure at 48 h. The width of the wound was measured and evaluated using the ImageJ software.^30^

#### Elisa

MDA MB-231 cells were treated for 48 h with NOC, **iMPZ-1**, and compounds **iMPZ-3**, **iMPZ-8**, and **iMPZ-12**. The cells were trypsinized and protein lysate extracted using RiPA buffer with a protease inhibitor. The BCA assay measured protein levels in each group. Protein levels were measured by direct ELISA using the required samples.^20^ The ELISA approach coated high affinity plates with β-tubulin antibody. After blocking with BSA, cell lysates and standards were added and kept at room temperature for an hour. Washing was done five times in a 5 × 5 grid. Next, biotinylated β-tubulin antibody was added to the samples and incubated at room temperature for 2 h. Washing was done five times in a five-by-five layout. HRP-Streptavidin was added and incubated at room temperature for 30 min. Five times, five by five, were washed. TMB was added and incubated for 30 min in the dark. Final absorbance values were measured at 450 nm using a plate reader after adding a stop solution.^31^ Compounds-induced cell morphological alterations were assessed using an inverted microscope. H&E staining was also used to analyze MDA-MB-231 cells’ morphology. In 6-well plates, 5 × 10^3^ cells were seeded per well on coverslips and treated with 3 μM of compounds (NOC, **iMPZ-1**, **iMPZ-3**, **iMPZ-8**, and **iMPZ-12**). The cells were fixed with 4% paraformaldehyde and rinsed with PBS. After washing, cells were stained with hematoxylin for 1 min and eosin for 25 s. Finally, coverslips with cells were rinsed with distilled water, mounted with mounting solution, and imaged with an Olympus BX53 microscope with a DP74 camera attachment. H&E staining images were evaluated for nucleus and cytoplasm changes.

#### Immunofluorescence (IF) Staining

MDA-MB-231 cells were seeded at a density of 5 × 10^4^ cells per well on round coverslips in a 6-well plate and subsequently treated with 3 μM NOC and **iMPZ-8**. Following compounds treatment, the cells underwent fixation with 4% paraformaldehyde for a duration of 15 min. Subsequently, they underwent three washes with PBS buffer. The cells were subsequently treated with a 0.25% Triton X-100 solution for 10 min at room temperature to enhance cell membrane permeability. Each well was stained with 5 μL of DAPI solution at a concentration of 1 mg/mL. Following staining, cell nuclei were imaged utilizing an Olympus BX53 fluorescence microscope with a DP74 camera attachment. The images were analyzed with the ImageJ software. Cells were seeded onto coverslips in 6-well plates at a density of 5 × 10^4^ cells per well and treated with 3 μM NOC and **iMPZ-8**. Cells were permeabilized by incubation in a 0.25% Triton X-100 solution at room temperature for 10 min. The cells were subsequently blocked using phosphate-buffered saline (PBS) with 5% bovine serum albumin (BSA) and incubated at room temperature for 1 h. Subsequently, the cells were incubated overnight at +4 °C with the anti-β-tubulin primary antibody at a dilution of 1:200 (2146S, Cell Signaling).^20^ The cells were washed three times with PBS buffer and then incubated with FITC-conjugated secondary antibody in a humidified chamber at room temperature for 1 h, followed by three additional washes with PBS buffer. The cells were subsequently mounted with a DAPI-containing medium and visualized using an Olympus BX53 fluorescence microscope with a DP74 camera attachment. Fluorescent images were analyzed utilizing ImageJ software (National Institutes of Health, Bethesda, MD).

#### Apoptosis Staining

MDA-MB-231 cells were exposed to incubation with 3 μM NOC and **iMPZ-8** for a duration of 48 h. Upon completion of incubation, cells were harvested using trypsinization, rinsed with cold PBS, and centrifuged to create a suspension. Thereafter, cells were stained with Annexin V-FITC and propidium iodide (PI) following the manufacturer’s guidelines and the approach outlined in the literature.^26^ The apoptosis-inducing efficacy of **iMPZ-8** and NOC treatment in MDA-MB-231 cells was quantitatively assessed using this method.

#### Cell Cycle Staining

Fifty thousand cells were inoculated into each well of a six-well plate. They were incubated with 3 μm NOC and **iMPZ-8** for a duration of 48 h. The cells were subsequently removed using trypsin, and the media was discarded. They were subsequently treated with 70% ethanol. Following incubation at −20 °C for 30 min, 1 μL of RNase was introduced and incubated for 5 min. Five microliters of propidium iodide (PI) dye were thereafter added and incubated for 15 min at room temperature in the absence of light. Following incubation, 200 μL of the staining solution was administered and analyzed using flow cytometry.^26^

## Supplementary Material



## Notes

Synthesized compounds (iMPZ1–15); ^1^H- and ^13^C NMR spectra; HRMS spectra of the compounds (PDF)
