# Supplementary material for: Novel Imidazopyridine–Oxadiazole β‑Tubulin Inhibitors Suppress Breast Cancer Migration and Induce Caspase-3-Mediated Apoptosis
Source: ACS Omega. 2026 Jan 2;11(2):3273–82. doi: 10.1021/acsomega.5c10113 (PMC12824745; doi:10.1021/acsomega.5c10113)
Supplement: Supplementary file 1 [file ao5c10113_si_001.pdf]

Novel Imidazopyridine–Oxadiazole β-Tubulin Inhibitors Suppress Breast Cancer Migration and Induce Caspase-3–Mediated Apoptosis

Mustafa Cakir[1], Burak Kuzu[2*]

[1]Department of Medical Biology, Faculty of Medicine, Van Yuzuncu Yil University, Türkiye

[2]Department of Pharmaceutical Chemistry, Faculty of Pharmacy, Van Yuzuncu Yil University, Türkiye

SUPPLEMENTARY INFORMATION

Table of Contents **Page**

**1.**	The synthesized compounds (**iMPZ1-15**)	2
**2.**	^{1}H and ^{13}C NMR spectra	3
**3.**	HRMS spectrum of the compounds	18

1. The synthesized compounds **(iMPZ1-15)**:

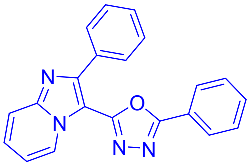

1

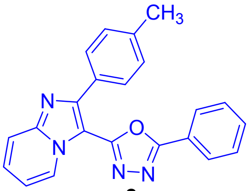

2

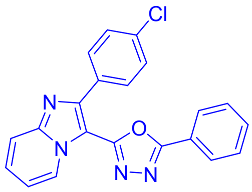

3

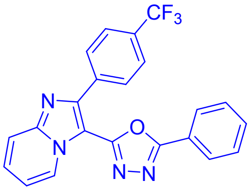

4

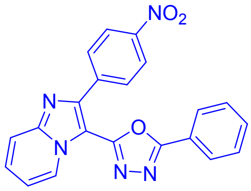

5

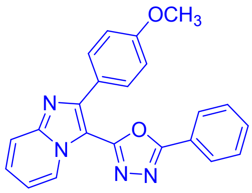

6

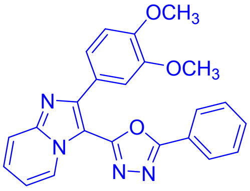

7

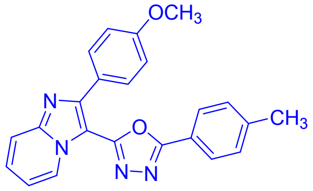

8

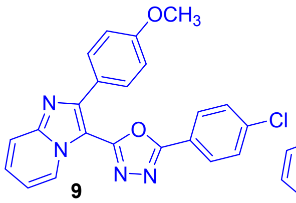

9

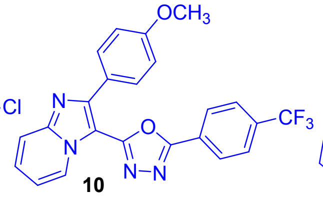

10

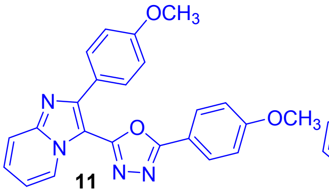

11

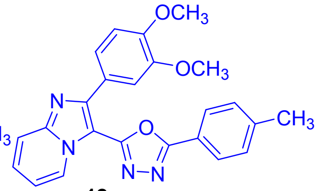

12

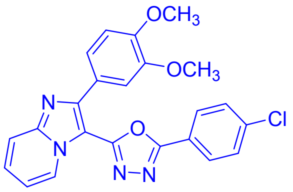

13

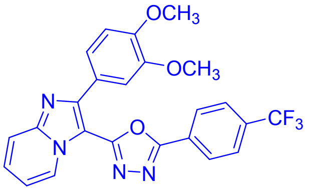

14

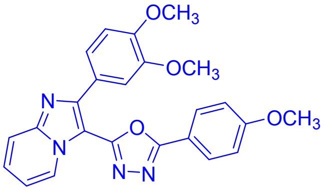

15

2. ^{1}H and ^{13}C NMR spectra

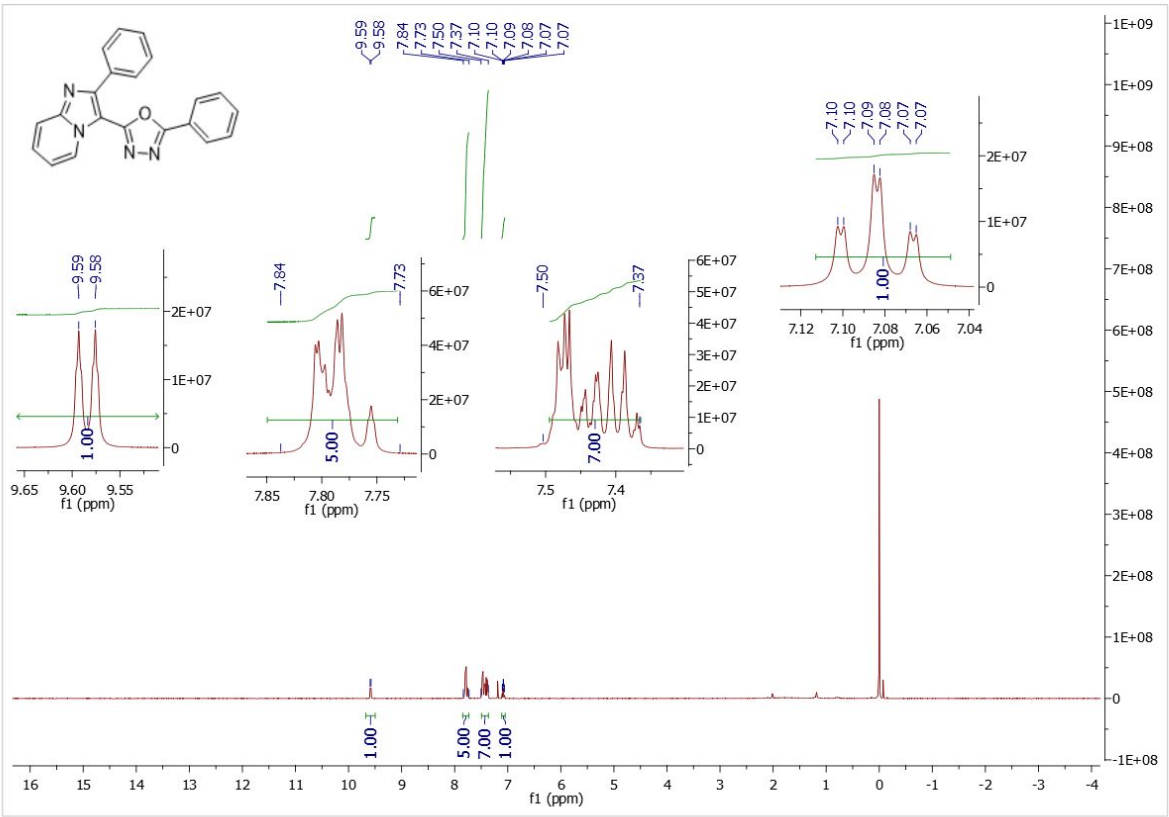

Figure S1. ^{1}H NMR spectrum of **iMPZ-1**

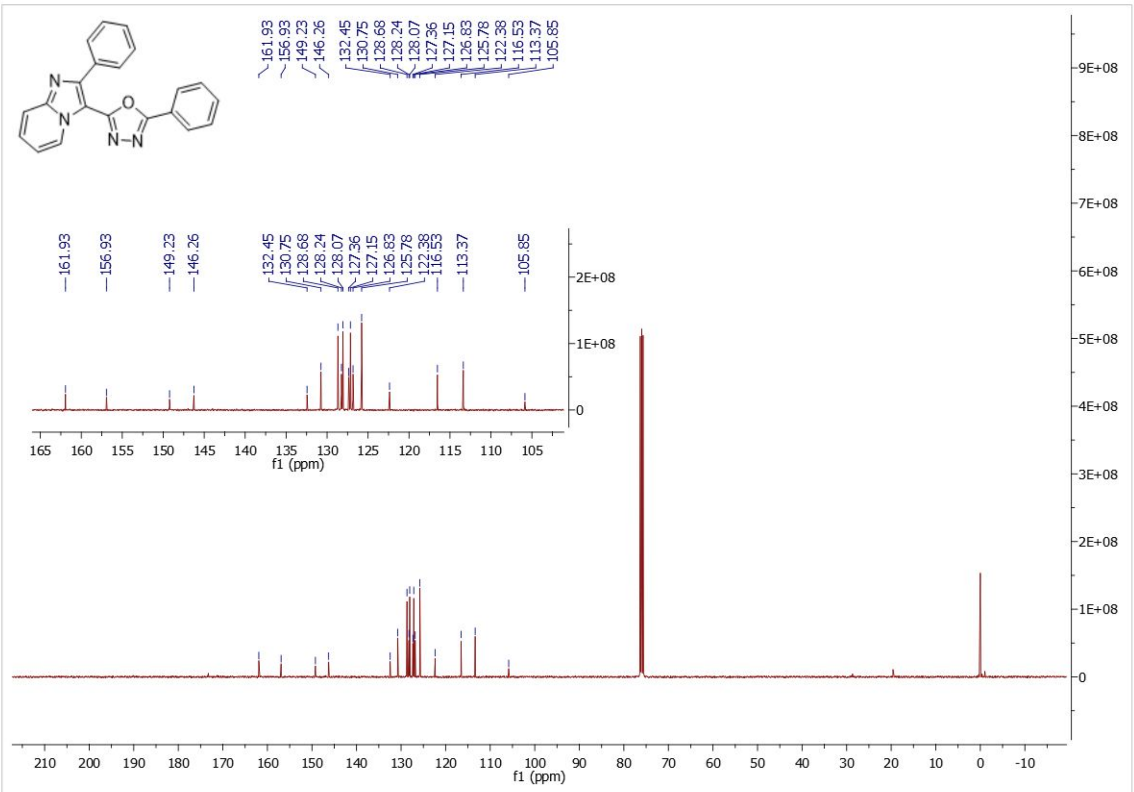

Figure S2. ^{13}C NMR spectrum of **iMPZ-1**

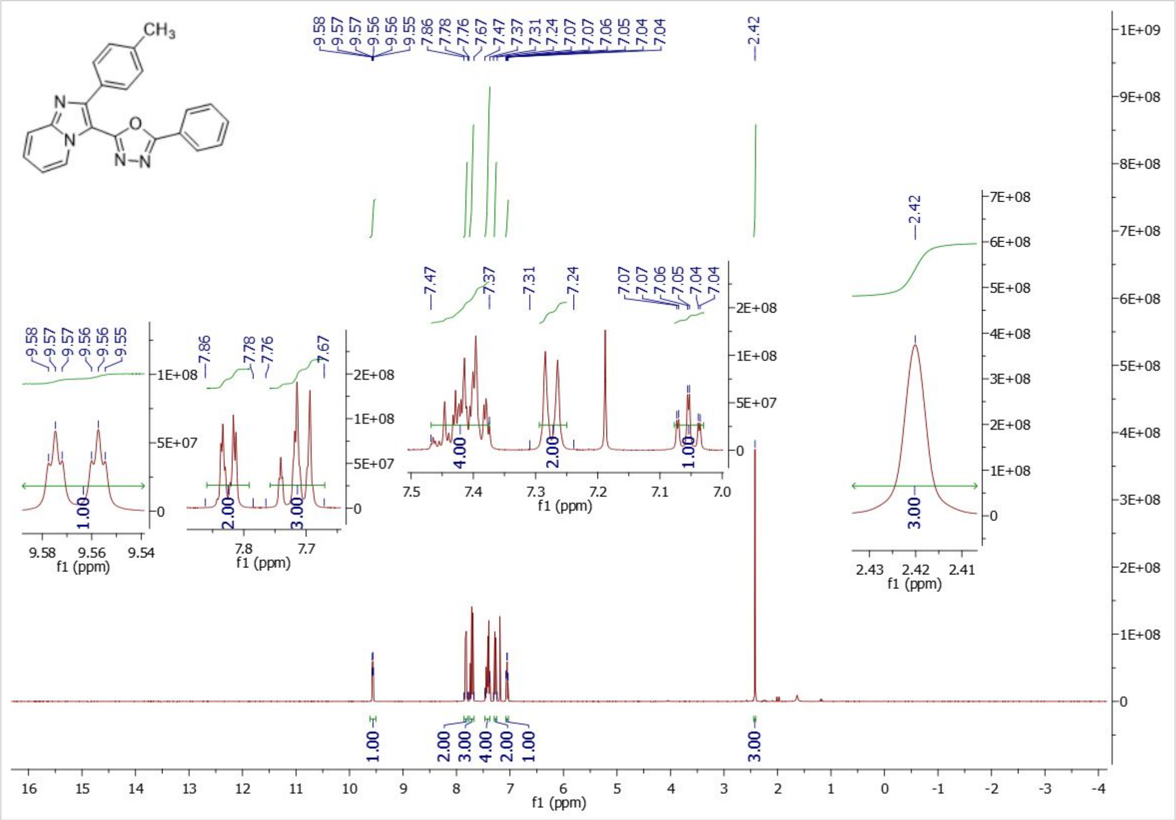

Figure S3. ^{1}H NMR spectrum of **iMPZ-2**

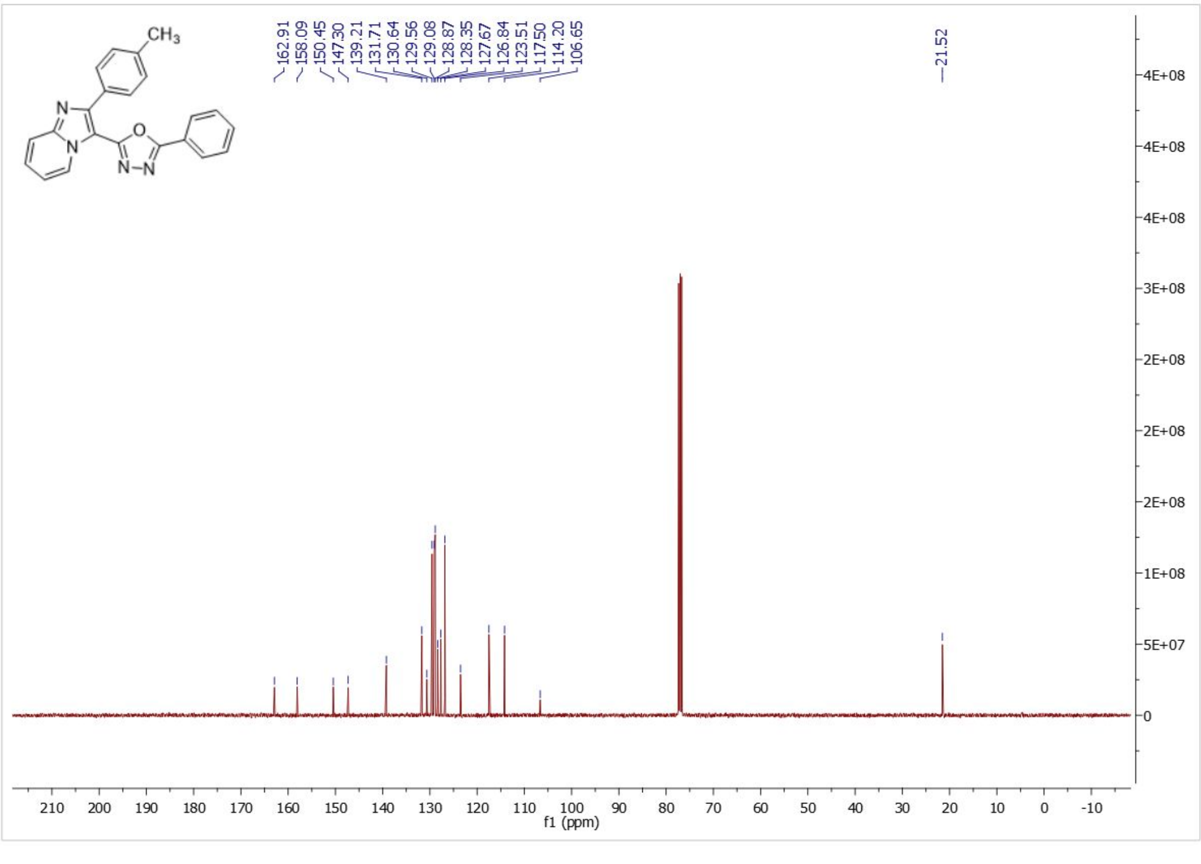

Figure S4. ^{13}C NMR spectrum of **iMPZ-2**

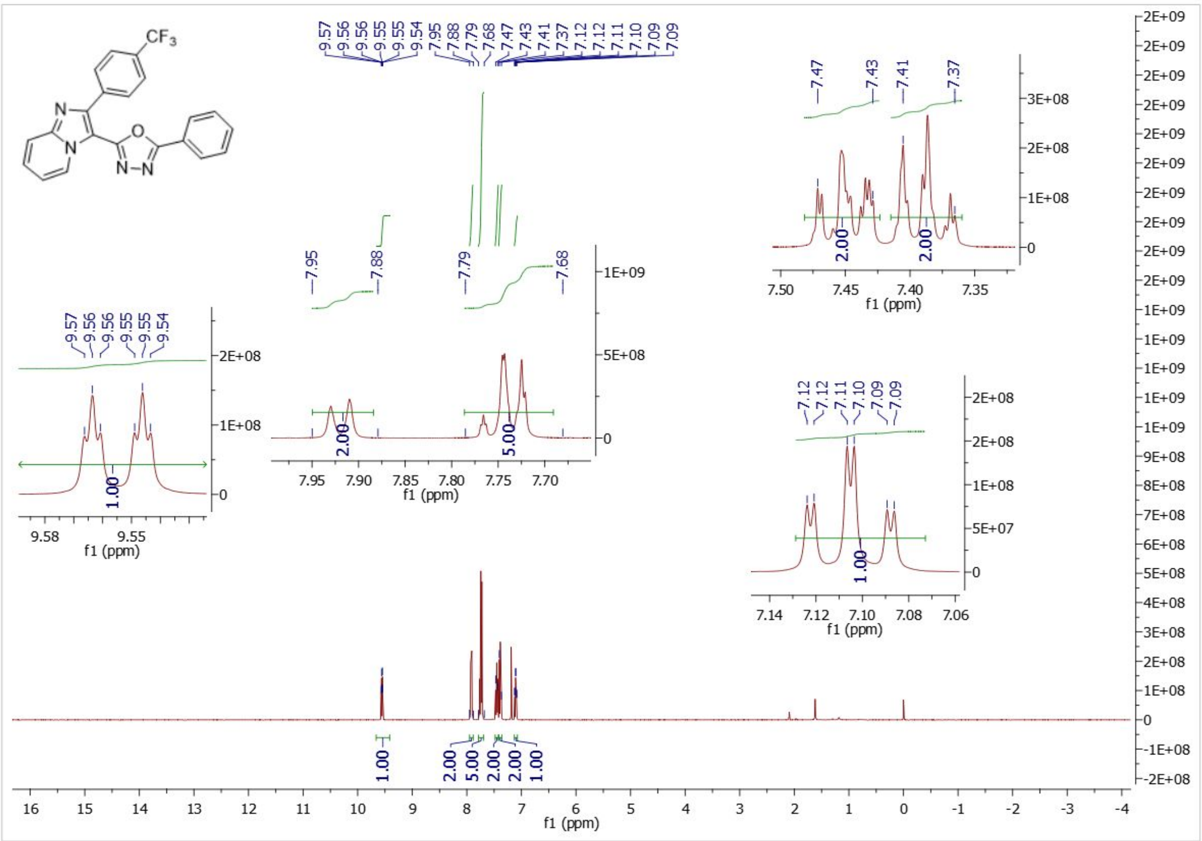

Figure S5. ^{1}H NMR spectrum of **iMPZ-3**

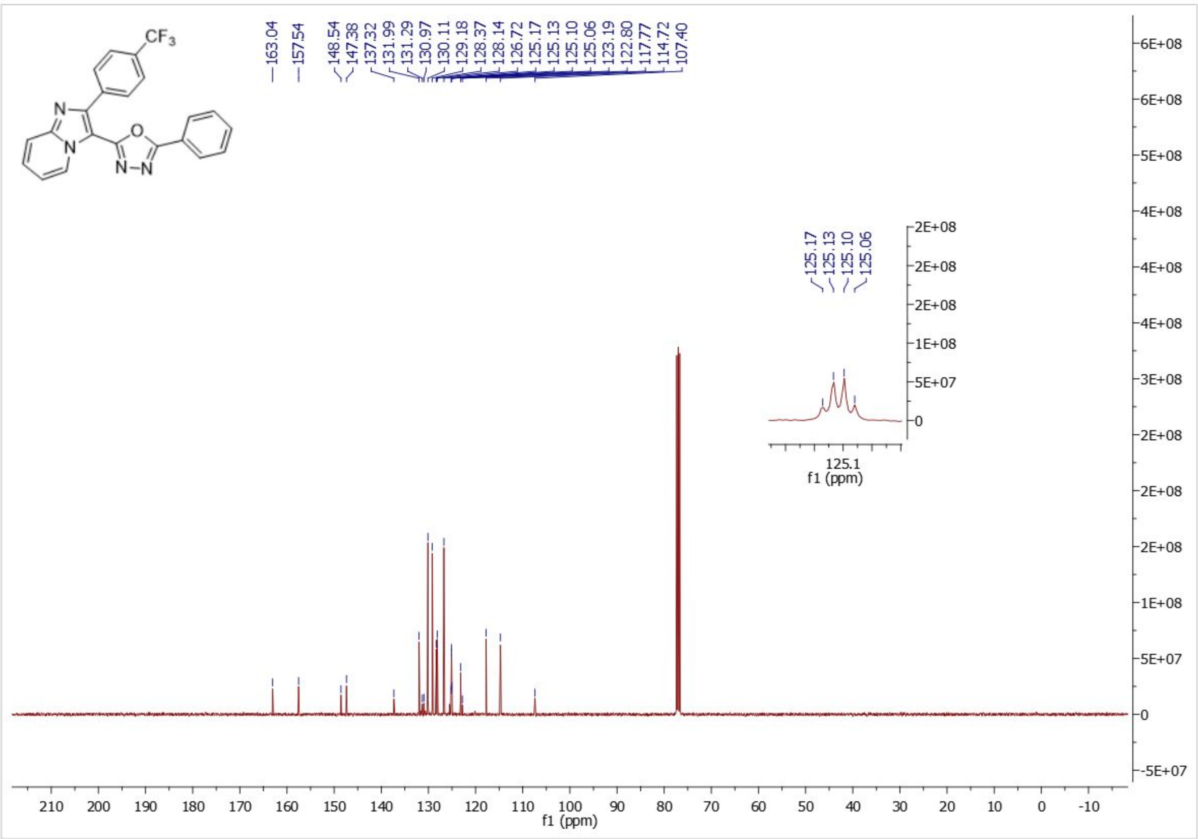

Figure S6. ^{13}C NMR spectrum of **iMPZ-3**

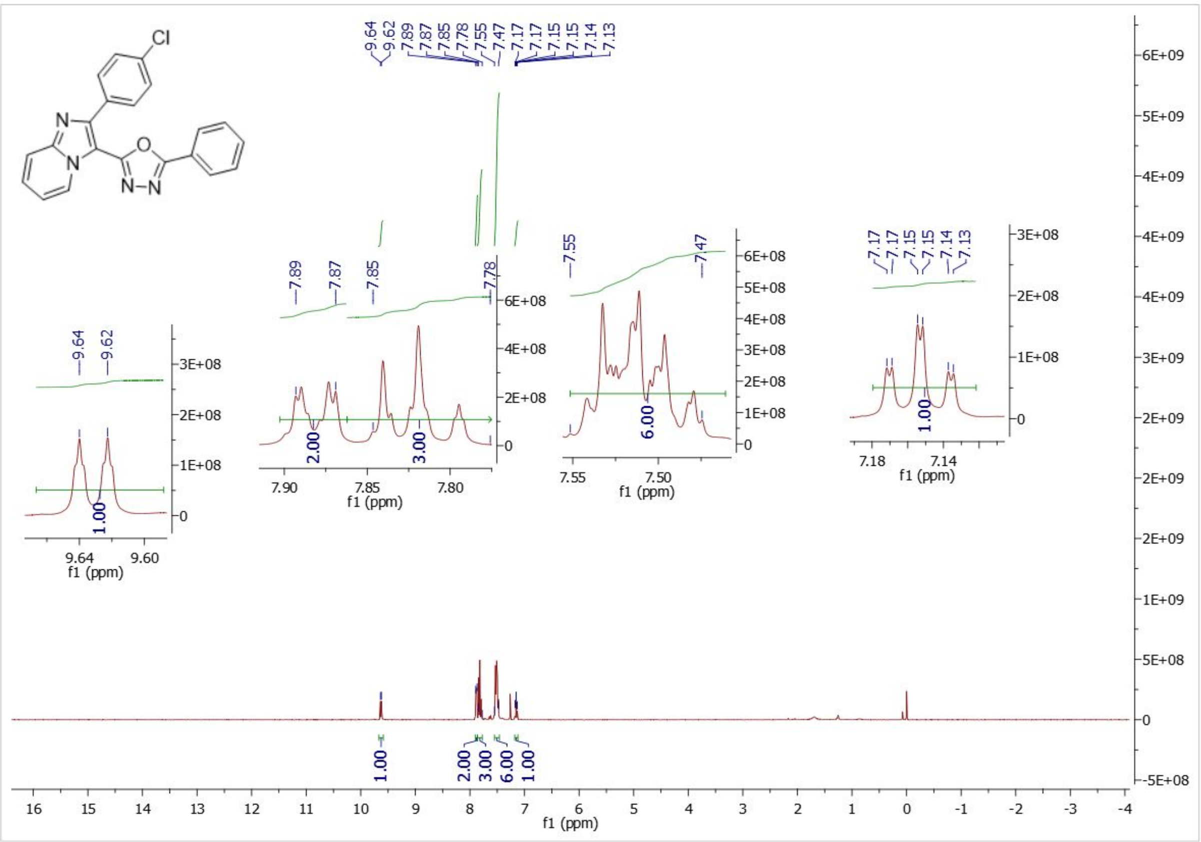

Figure S7. ^{1}H NMR spectrum of **iMPZ-4**

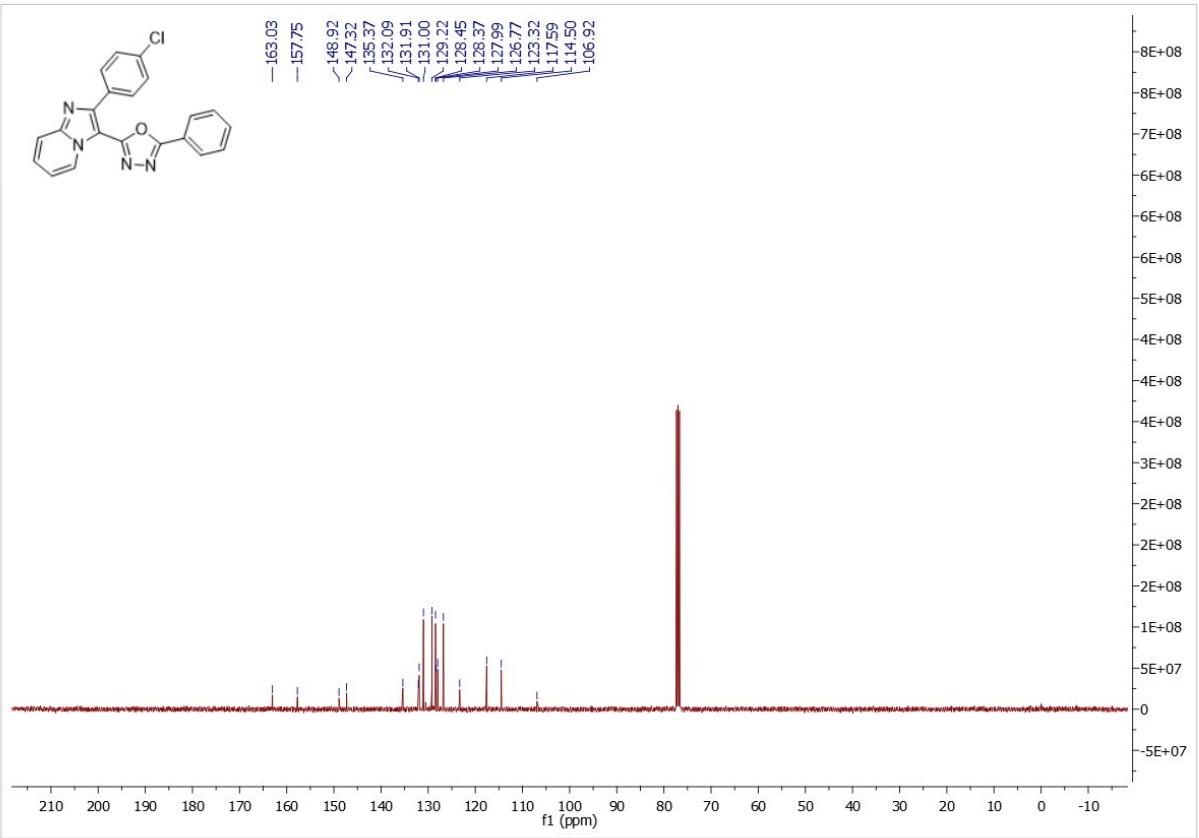

Figure S8. ^{13}C NMR spectrum of **iMPZ-4**

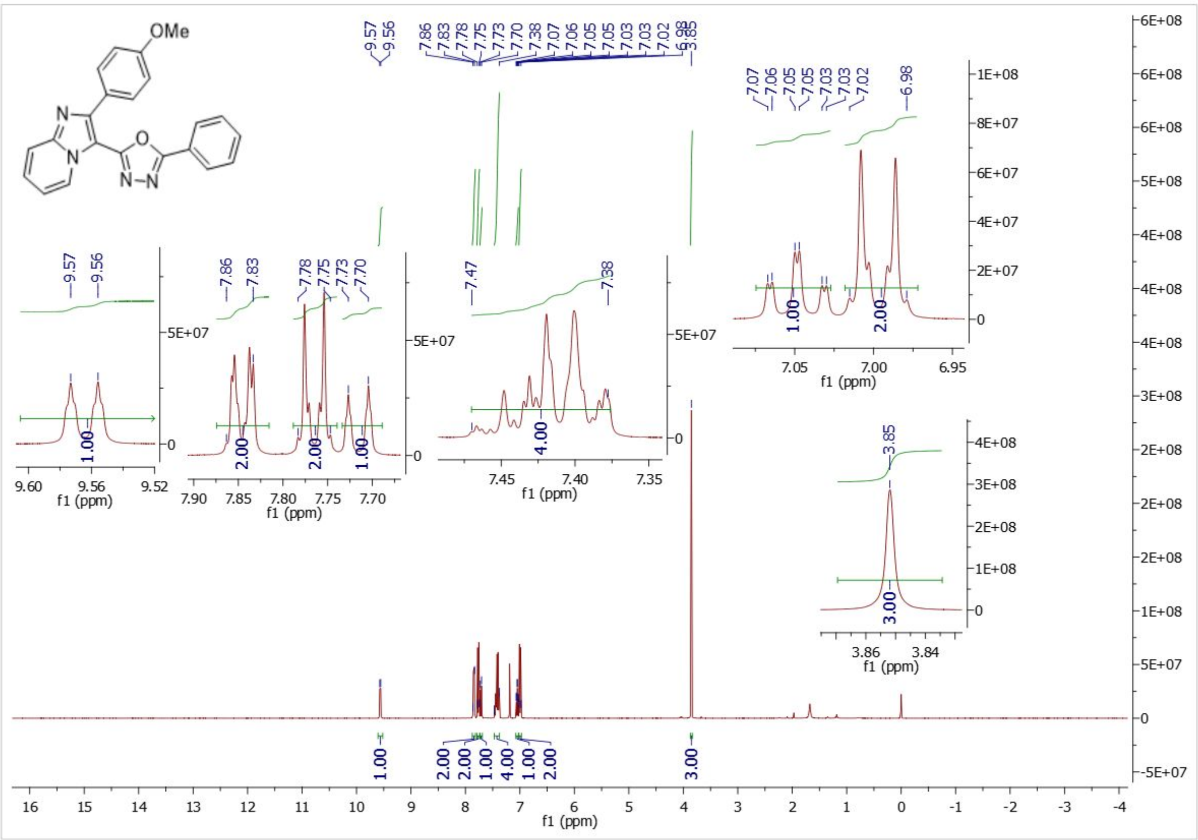

Figure S9. ^{1}H NMR spectrum of **iMPZ-5**

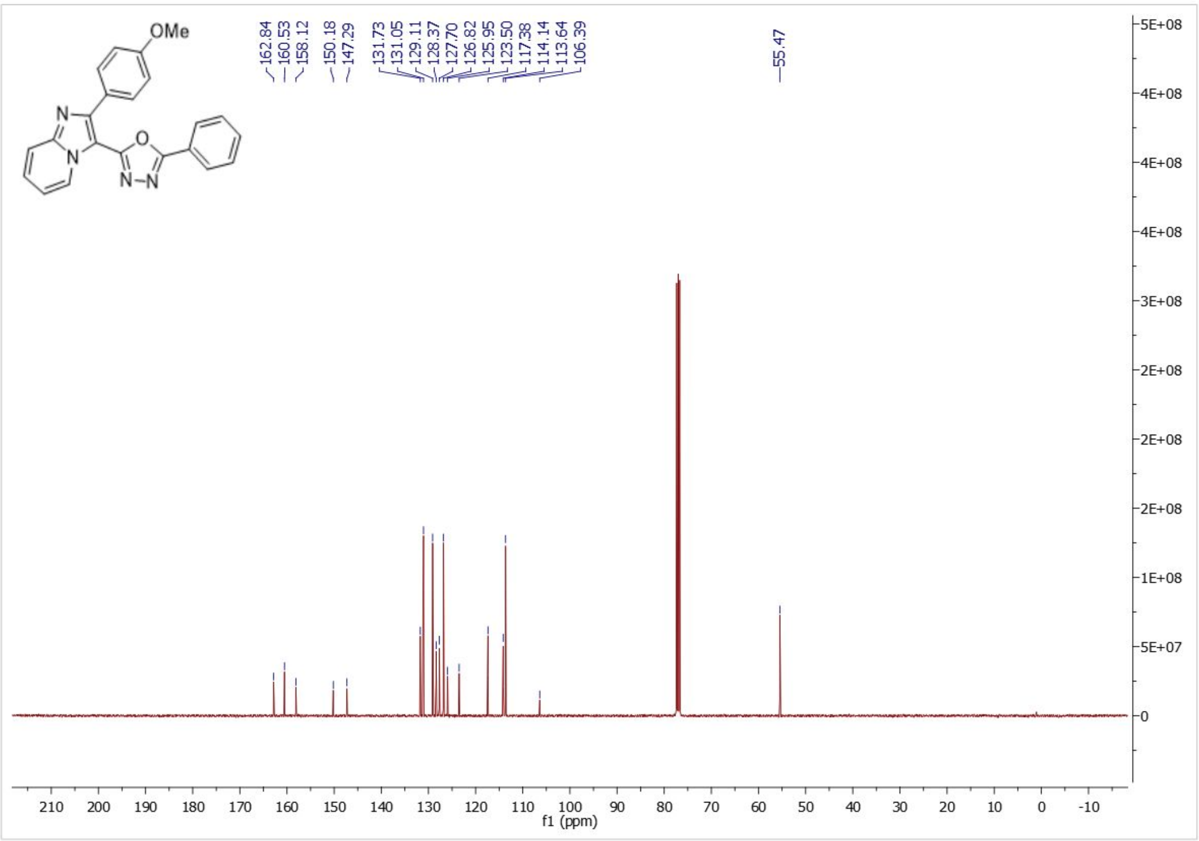

Figure S10. ^{13}C NMR spectrum of **iMPZ-5**

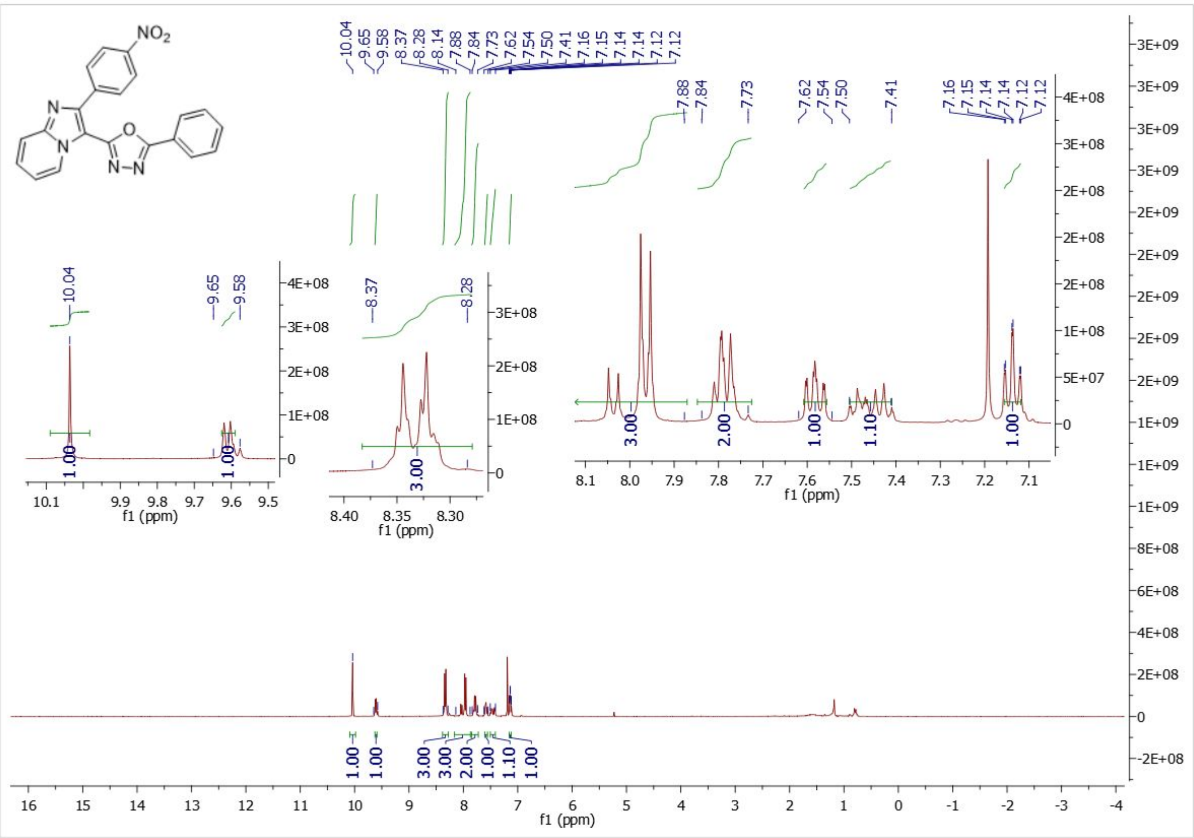

Figure S11. ^{1}H NMR spectrum of **iMPZ-6**

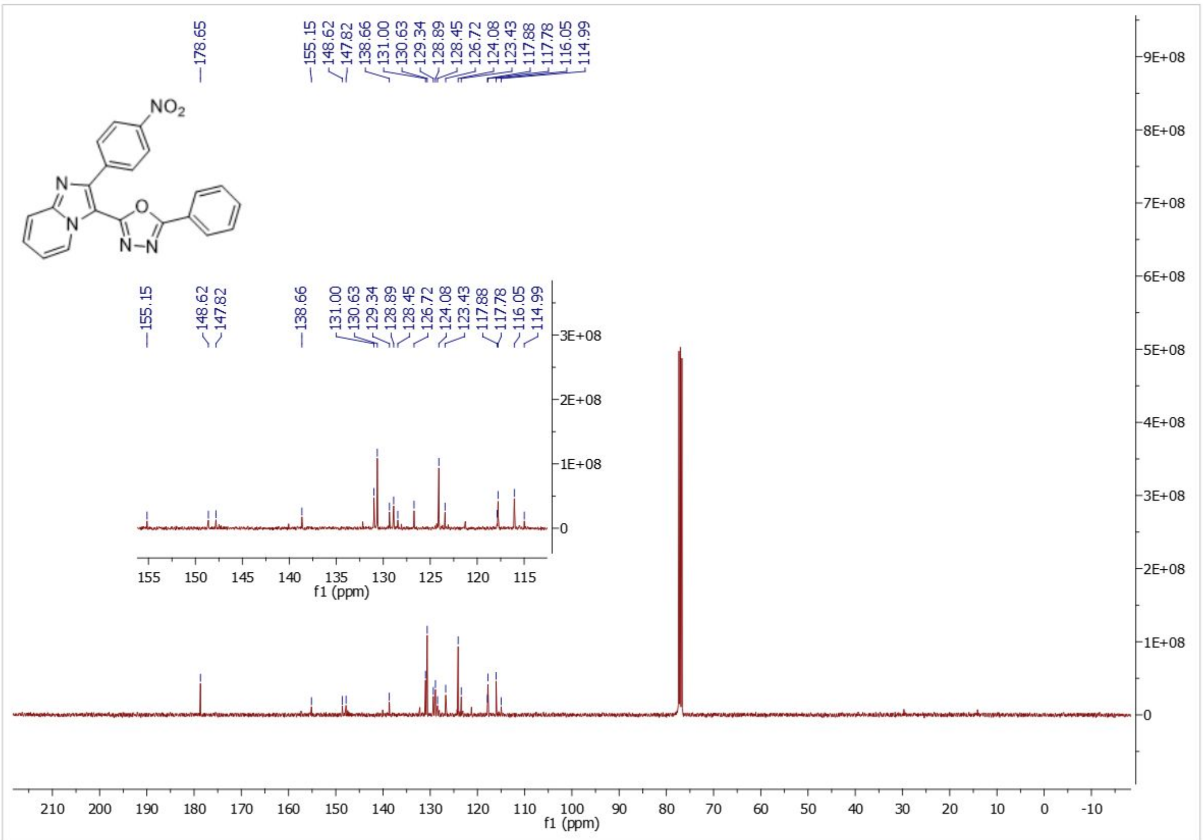

Figure S12. ^{13}C NMR spectrum of **iMPZ-6**

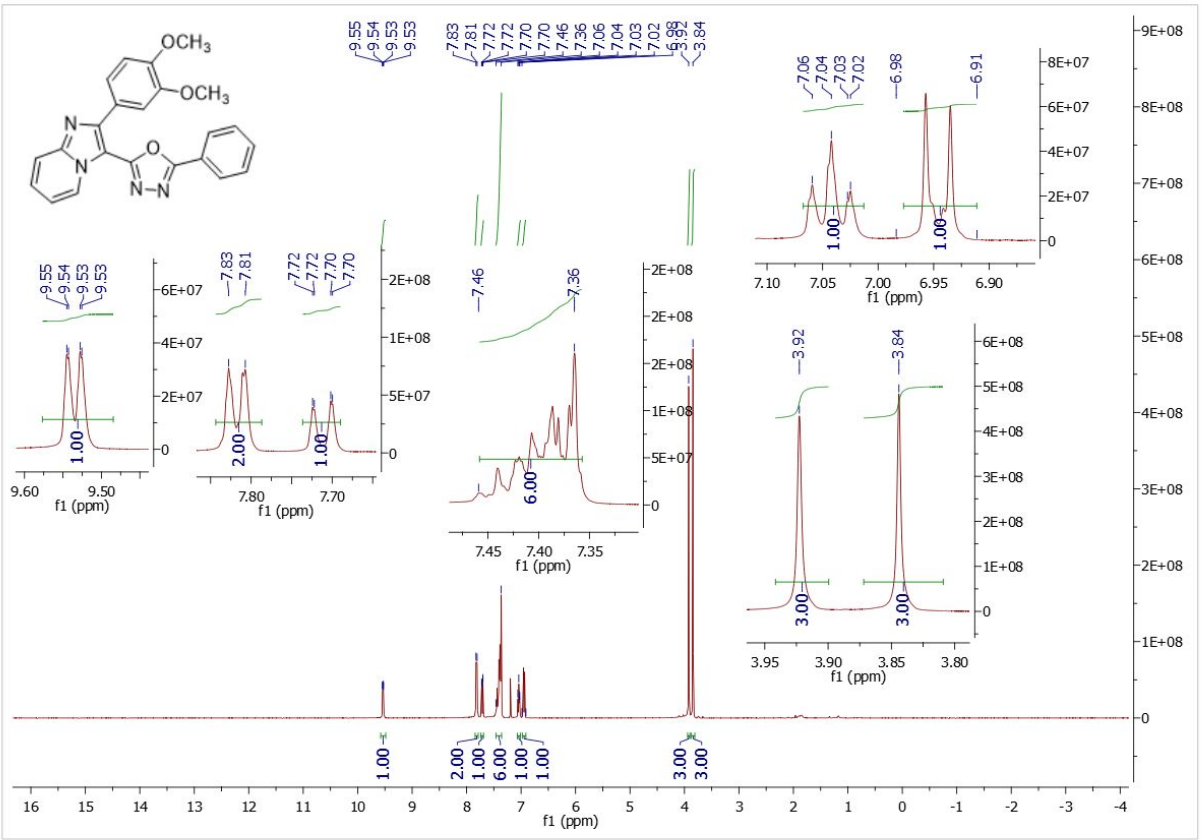

Figure S13. ^{1}H NMR spectrum of **iMPZ-7**

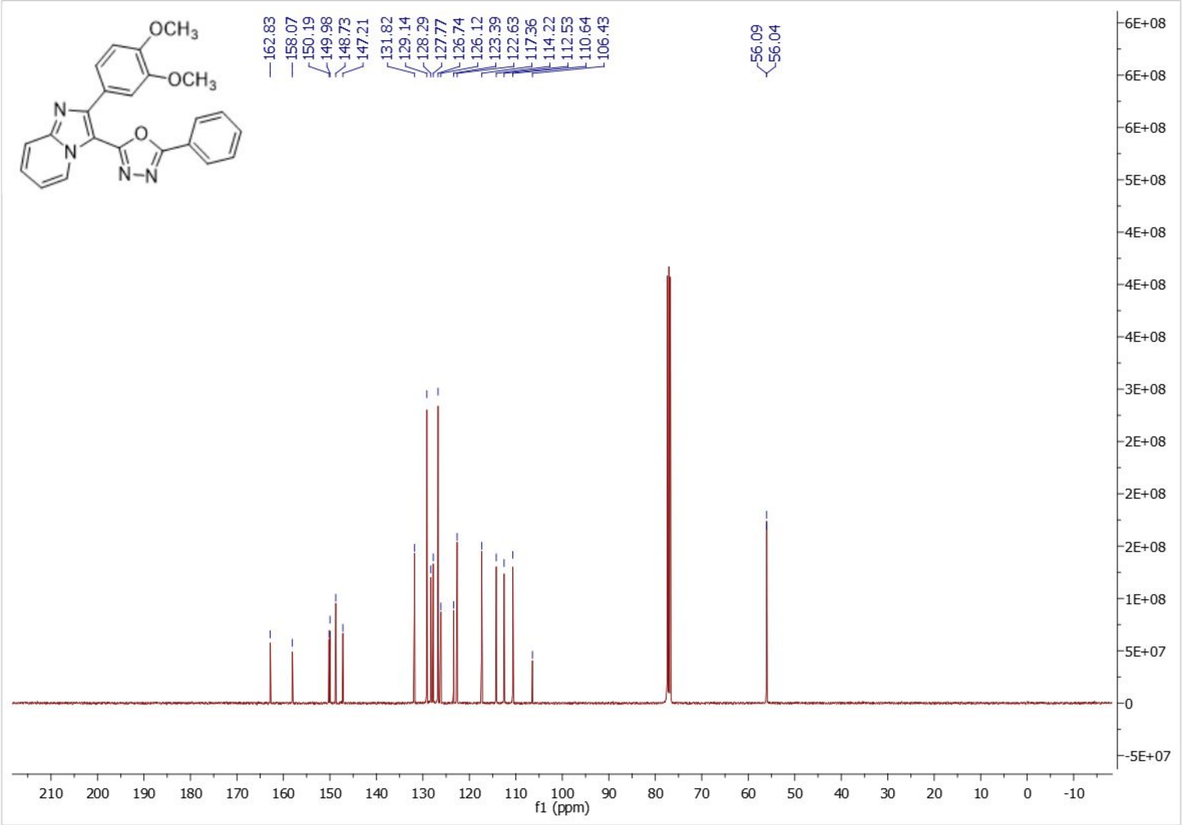

Figure S14. ^{13}C NMR spectrum of **iMPZ-7**

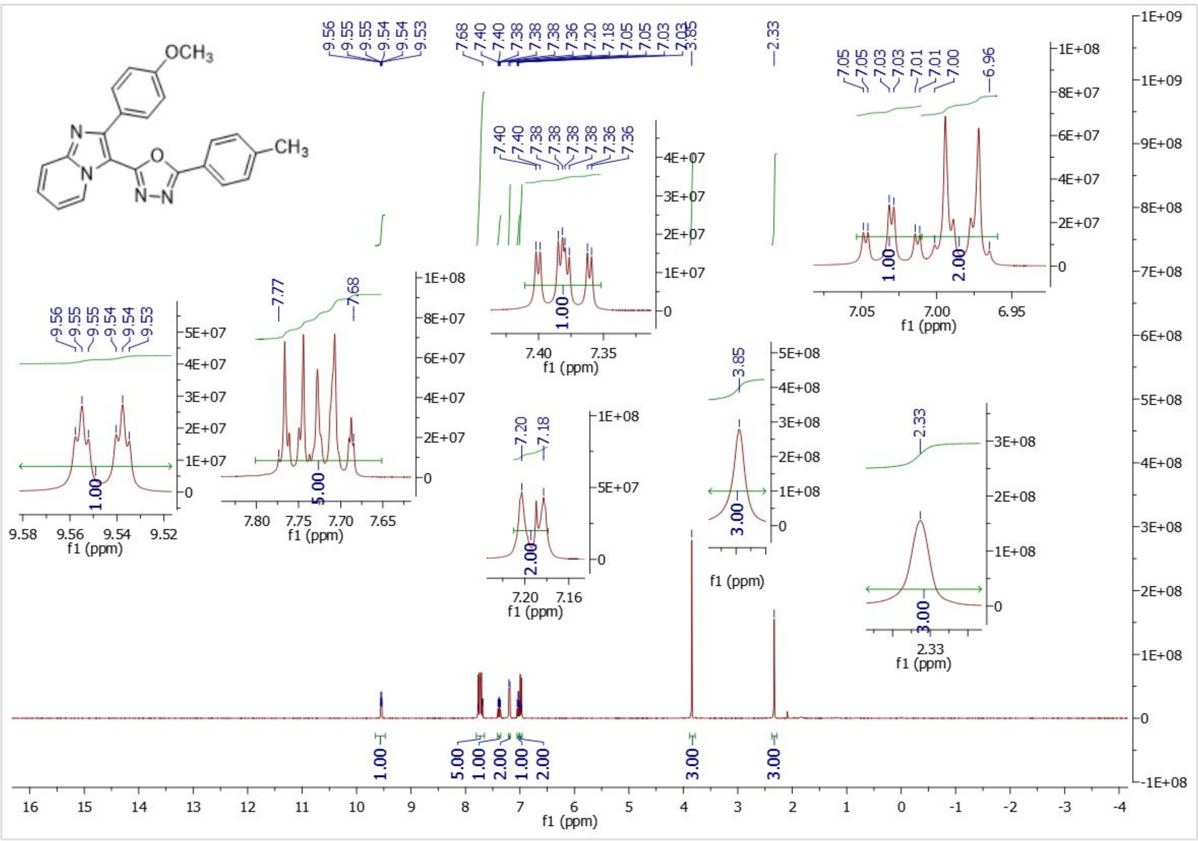

Figure S15. ^{1}H NMR spectrum of **iMPZ-8**

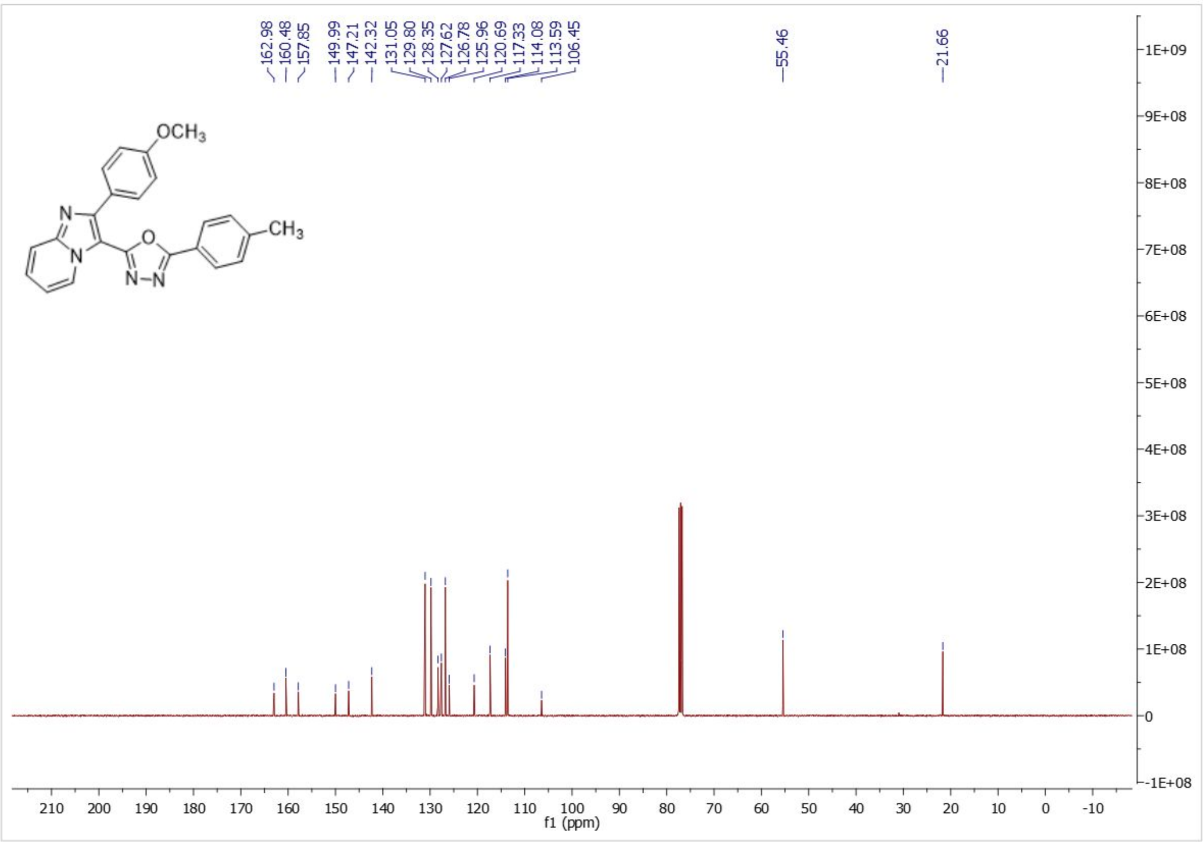

Figure S16. ^{13}C NMR spectrum of **iMPZ-8**

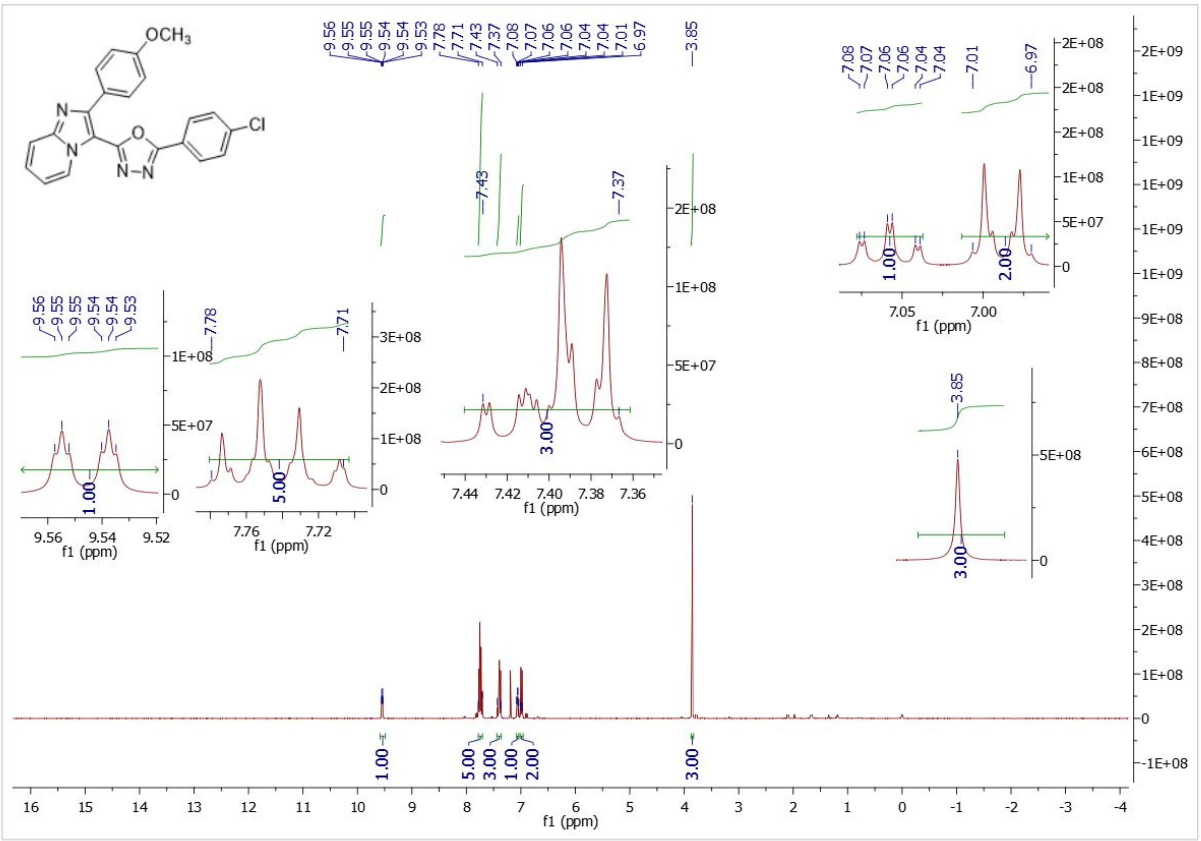

Figure S17. ^{1}H NMR spectrum of **iMPZ-9**

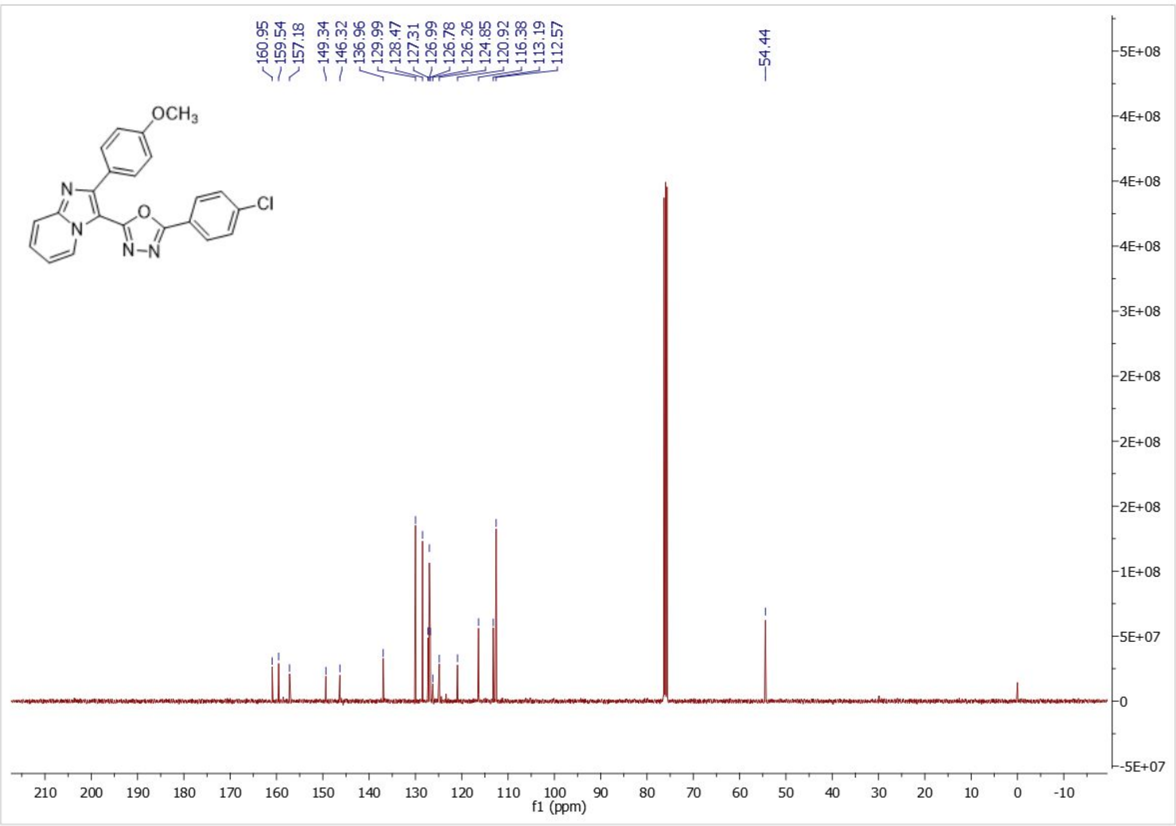

Figure S18. ^{13}C NMR spectrum of **iMPZ-9**

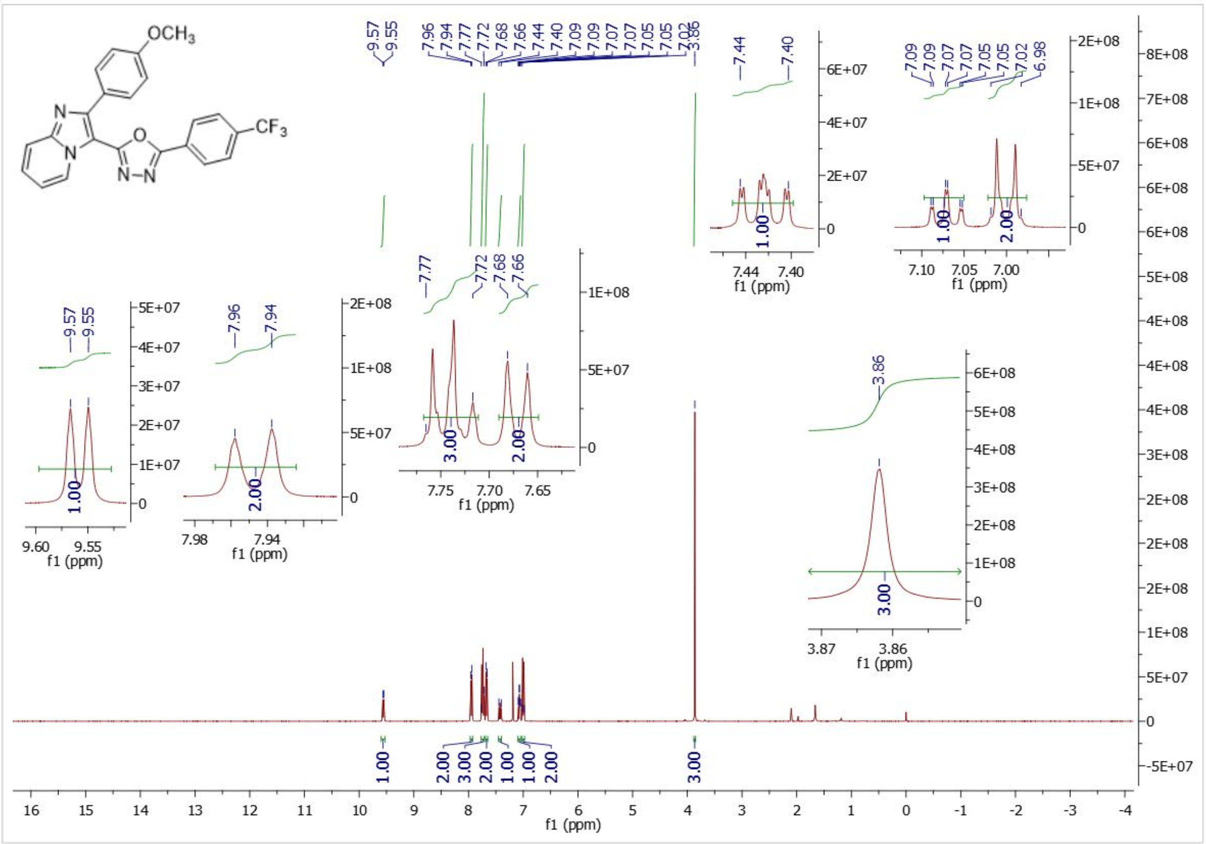

Figure S19. ^{1}H NMR spectrum of **iMPZ-10**

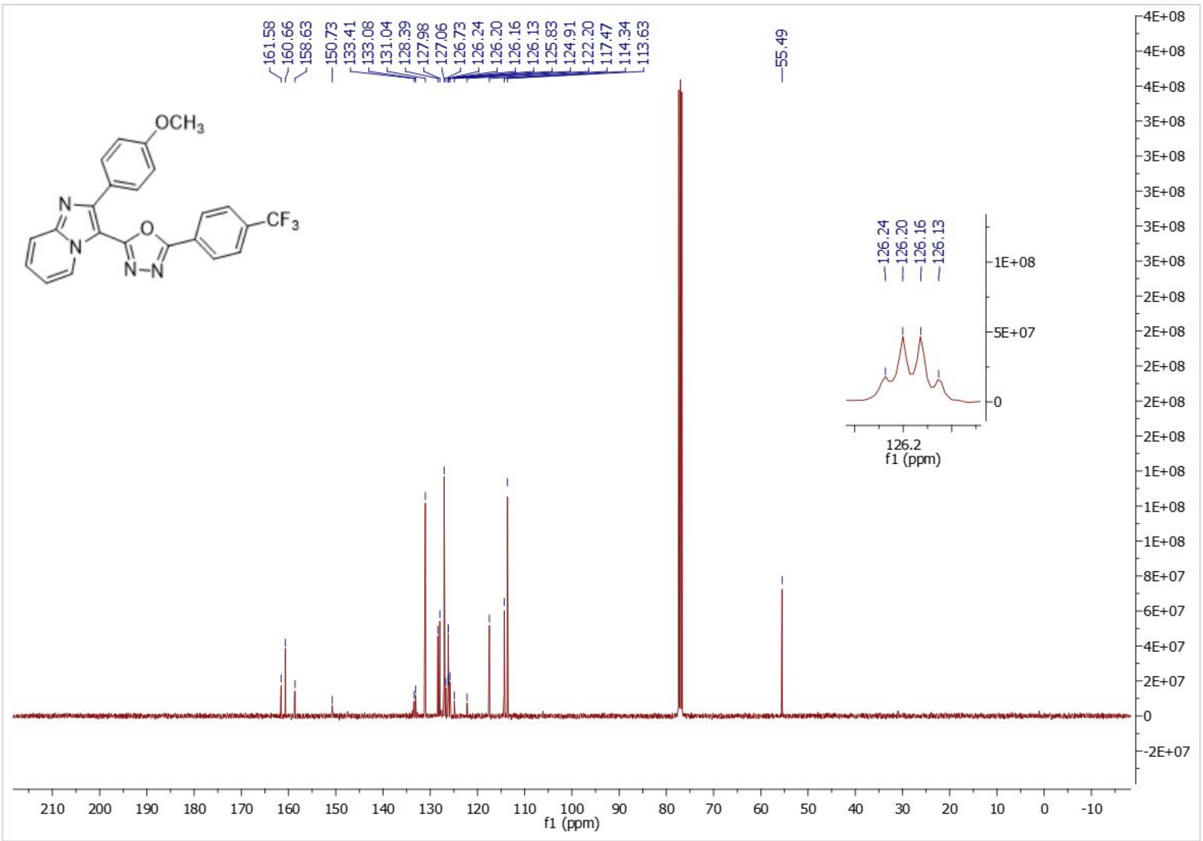

Figure S20. ^{13}C NMR spectrum of **iMPZ-10**

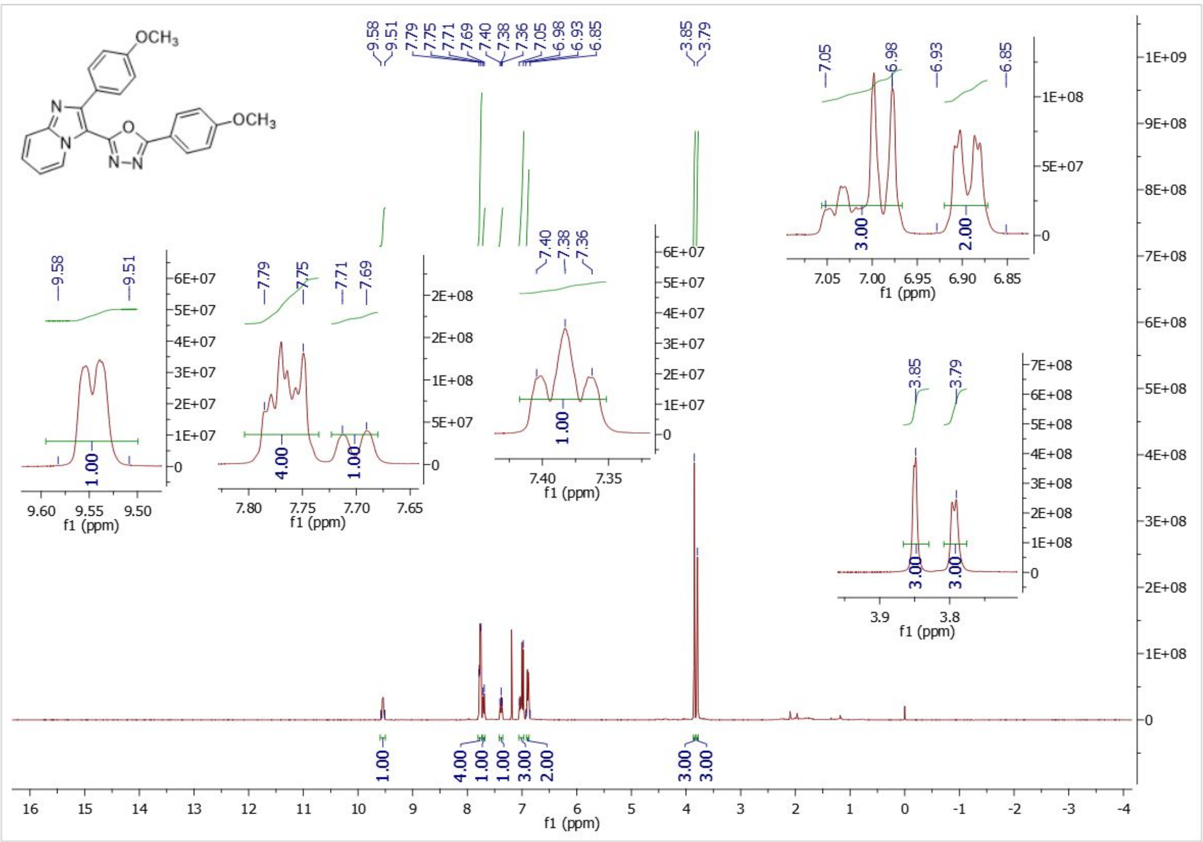

Figure S21. ^{1}H NMR spectrum of **iMPZ-11**

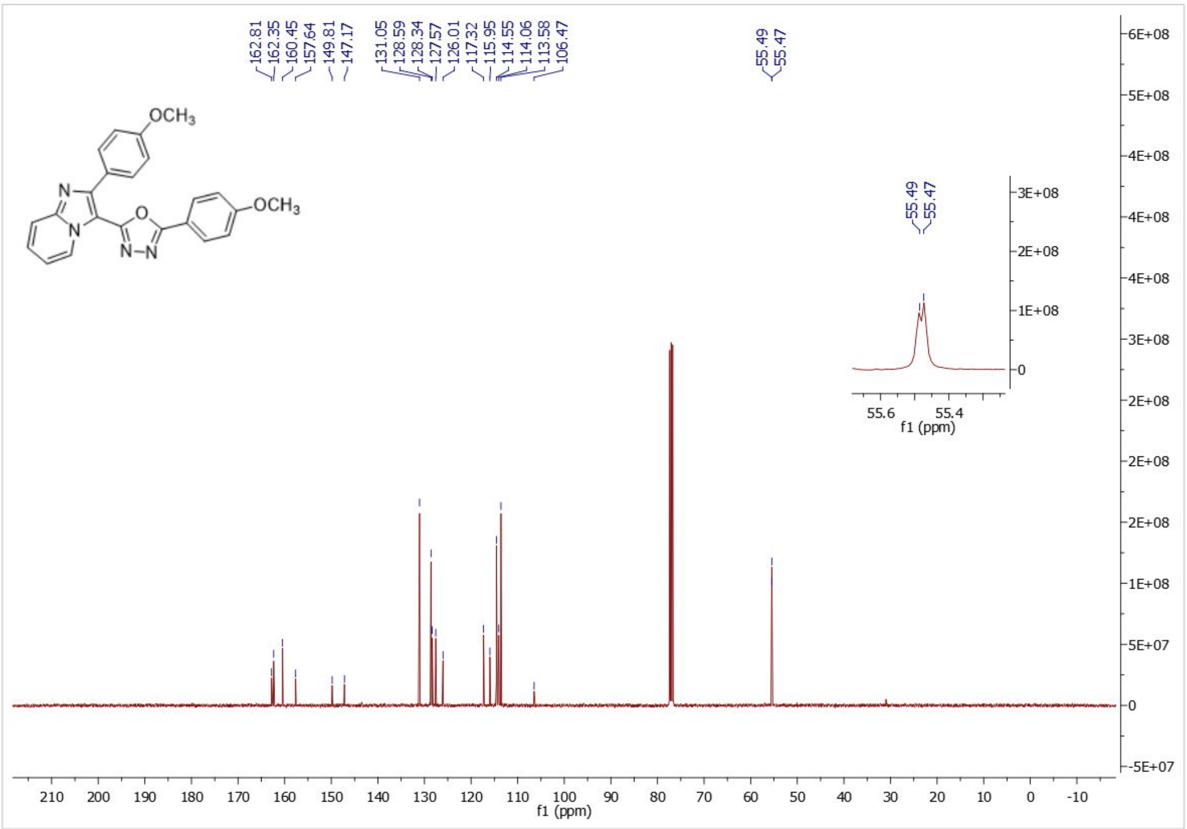

Figure S22. ^{13}C NMR spectrum of **iMPZ-11**

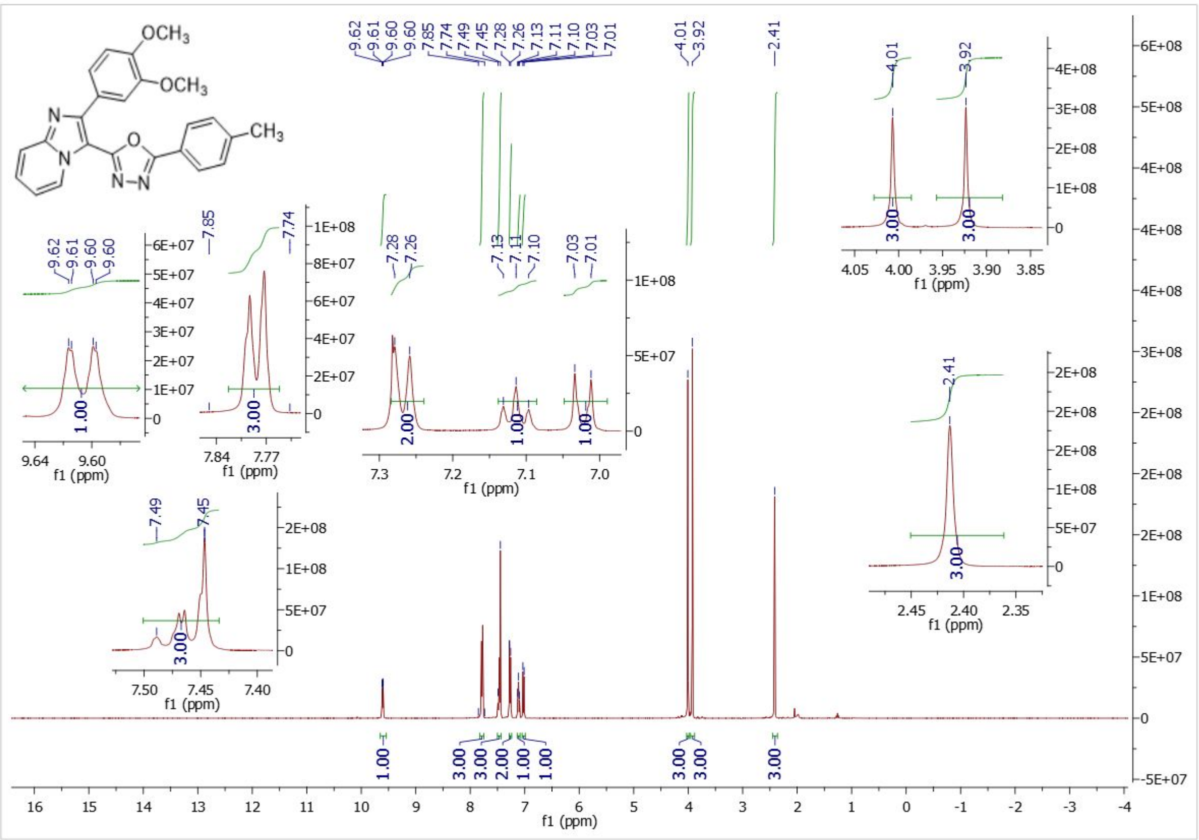

Figure S23. ^{1}H NMR spectrum of **iMPZ-12**

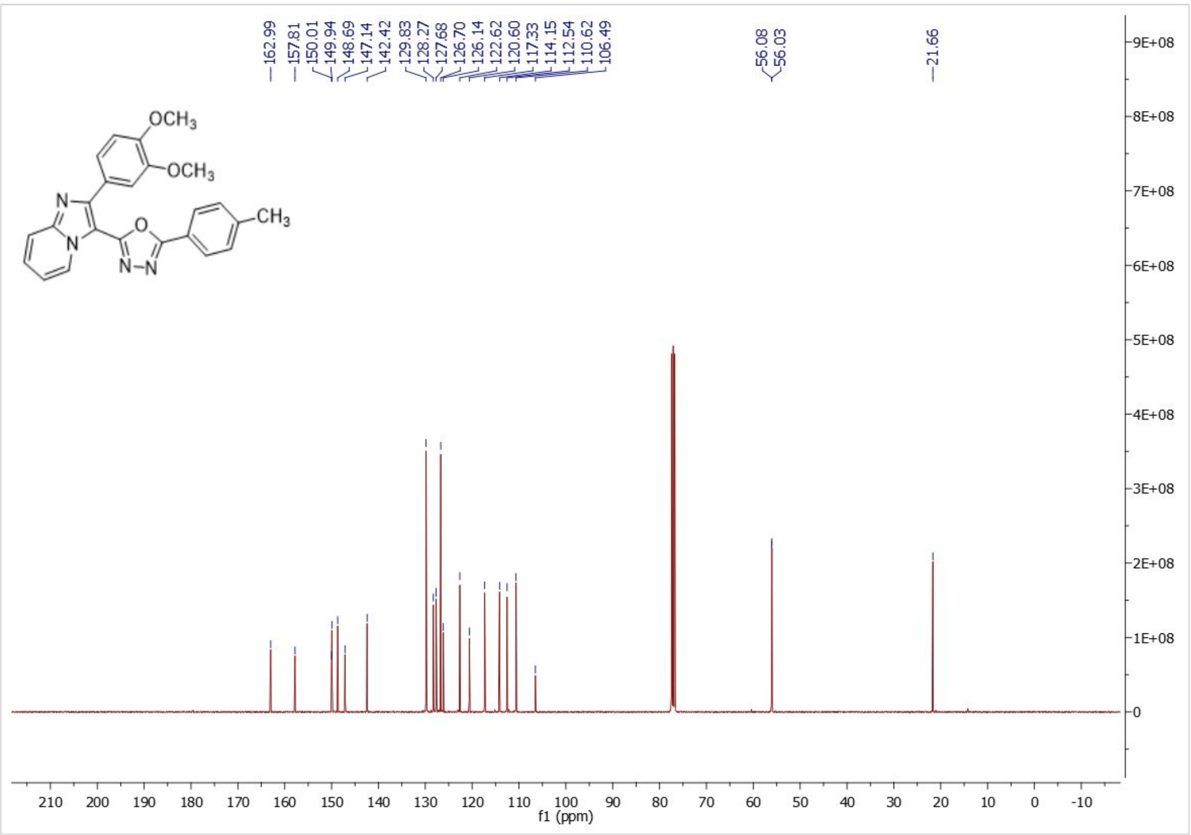

Figure S24. ^{13}C NMR spectrum of **iMPZ-12**

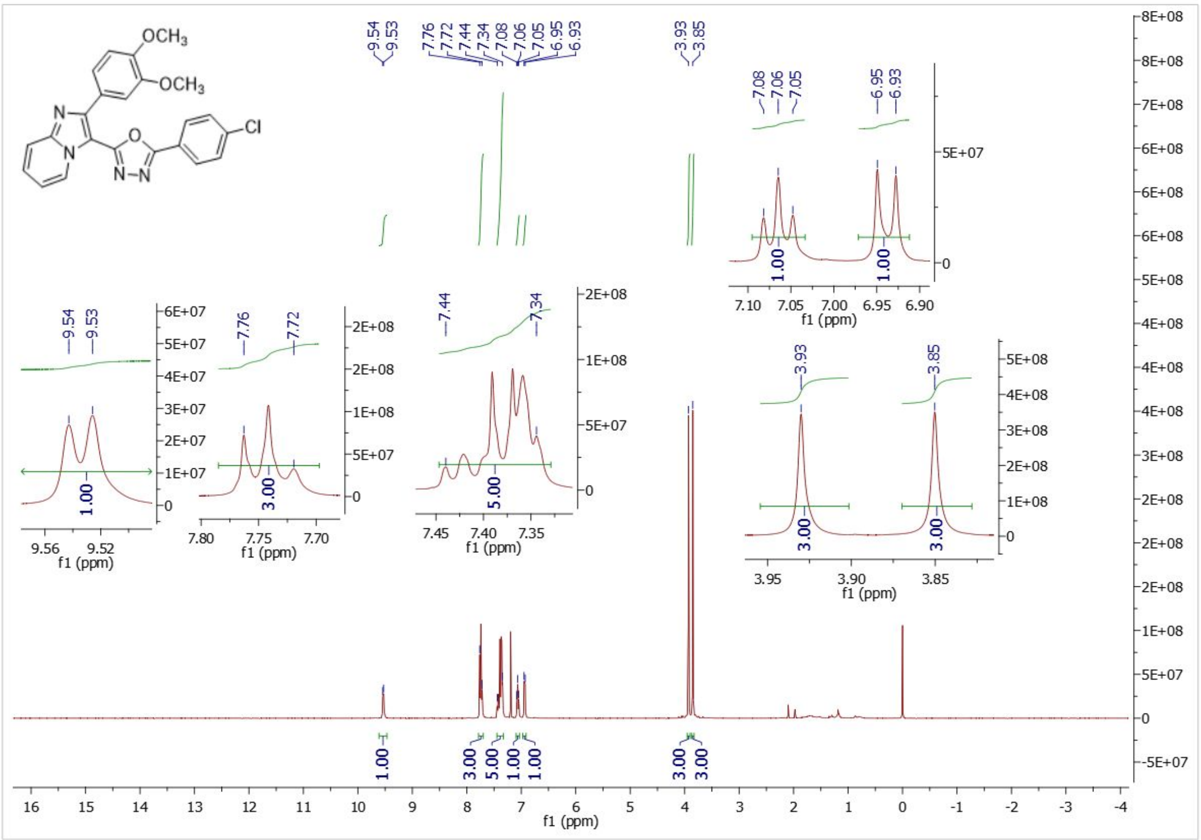

Figure S25. ^{1}H NMR spectrum of **iMPZ-13**

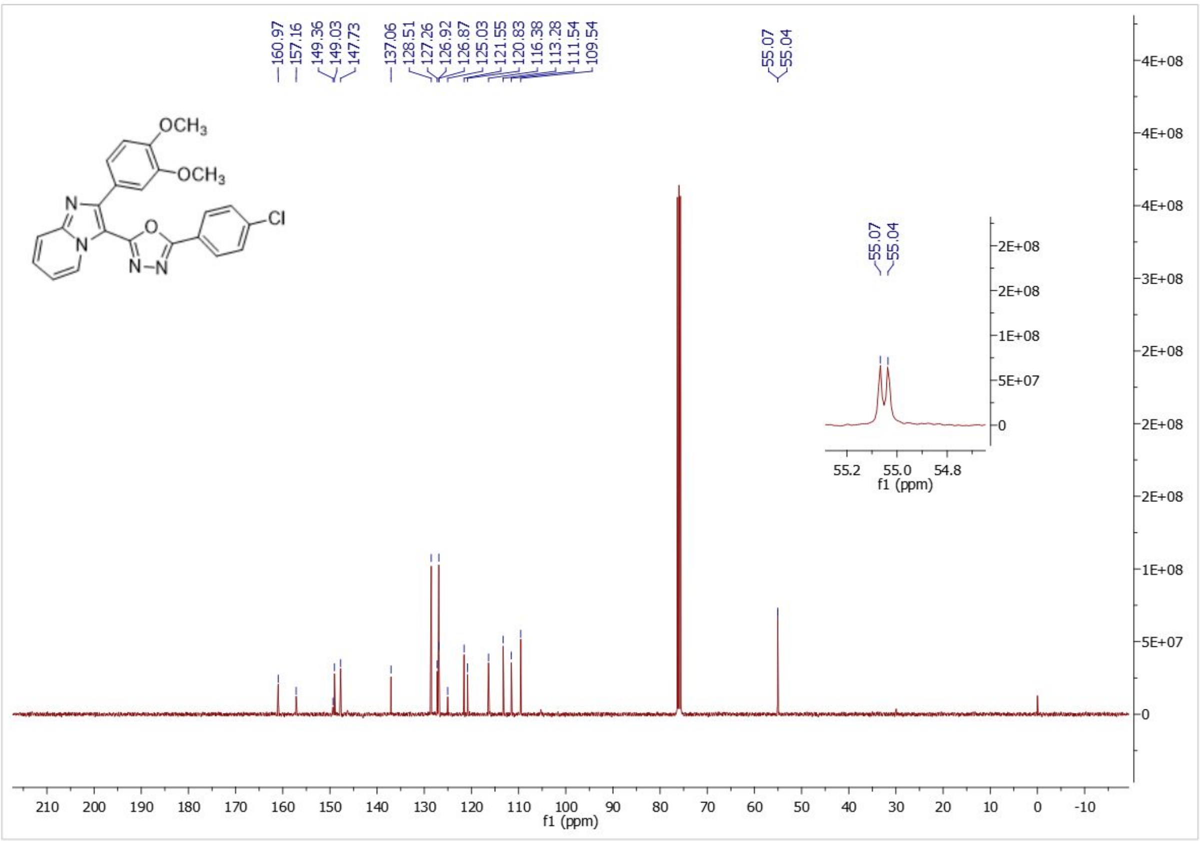

Figure S26. ^{13}C NMR spectrum of **iMPZ-13**

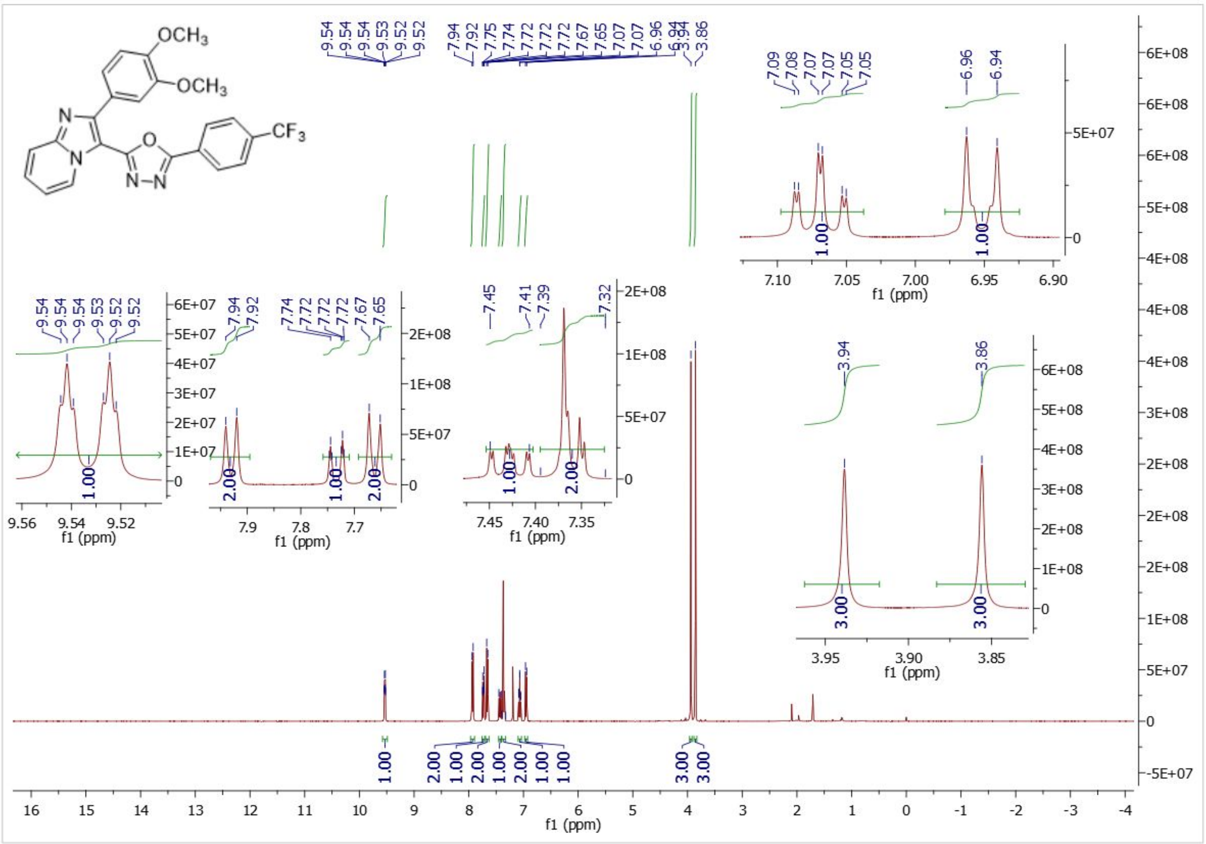

Figure S27. ^{1}H NMR spectrum of **iMPZ-14**

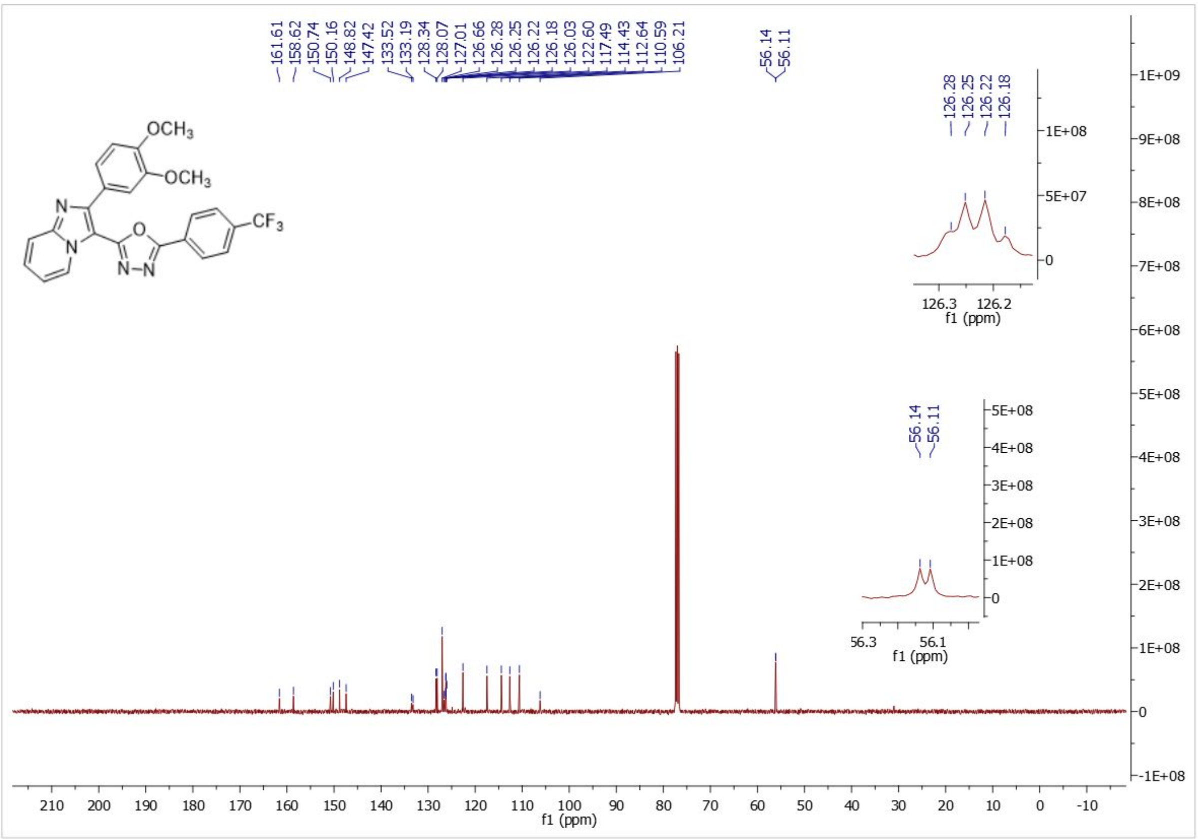

Figure S28. ^{13}C NMR spectrum of **iMPZ-14**

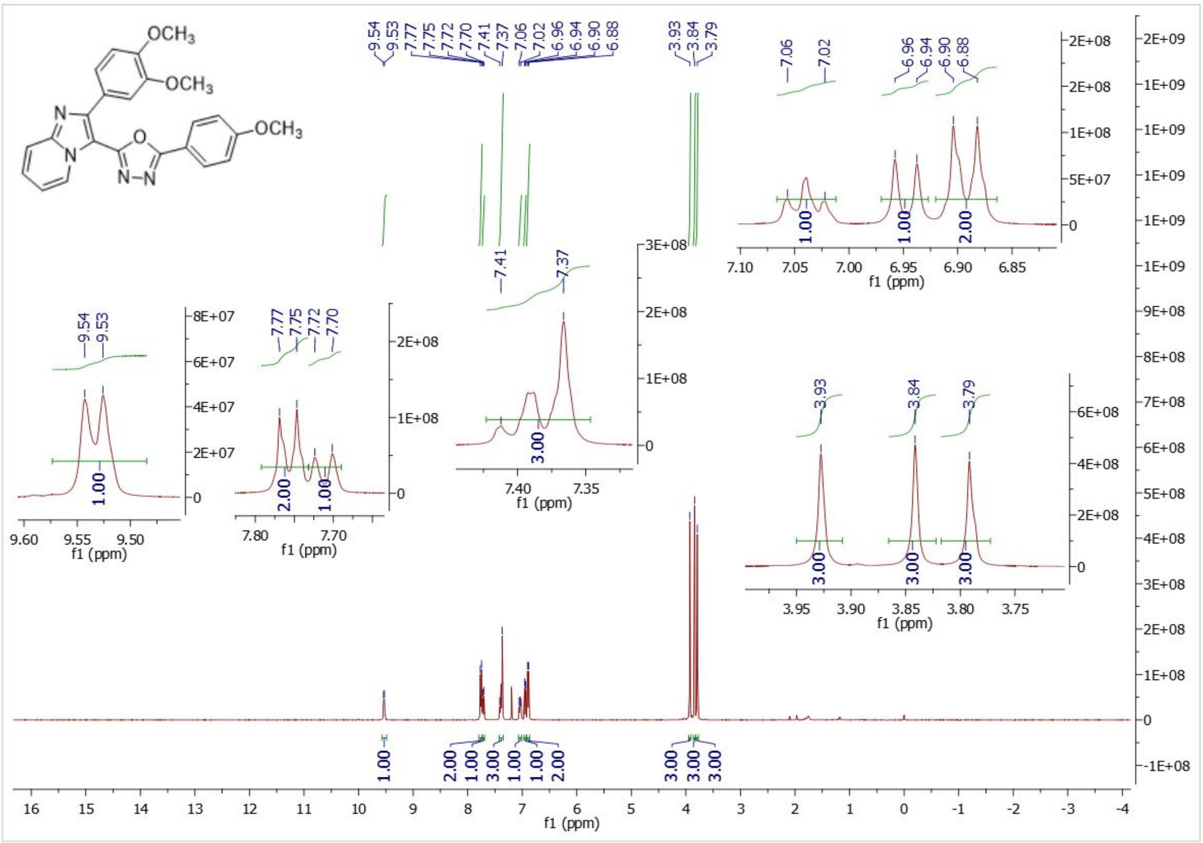

Figure S29. ^{1}H NMR spectrum of **iMPZ-15**

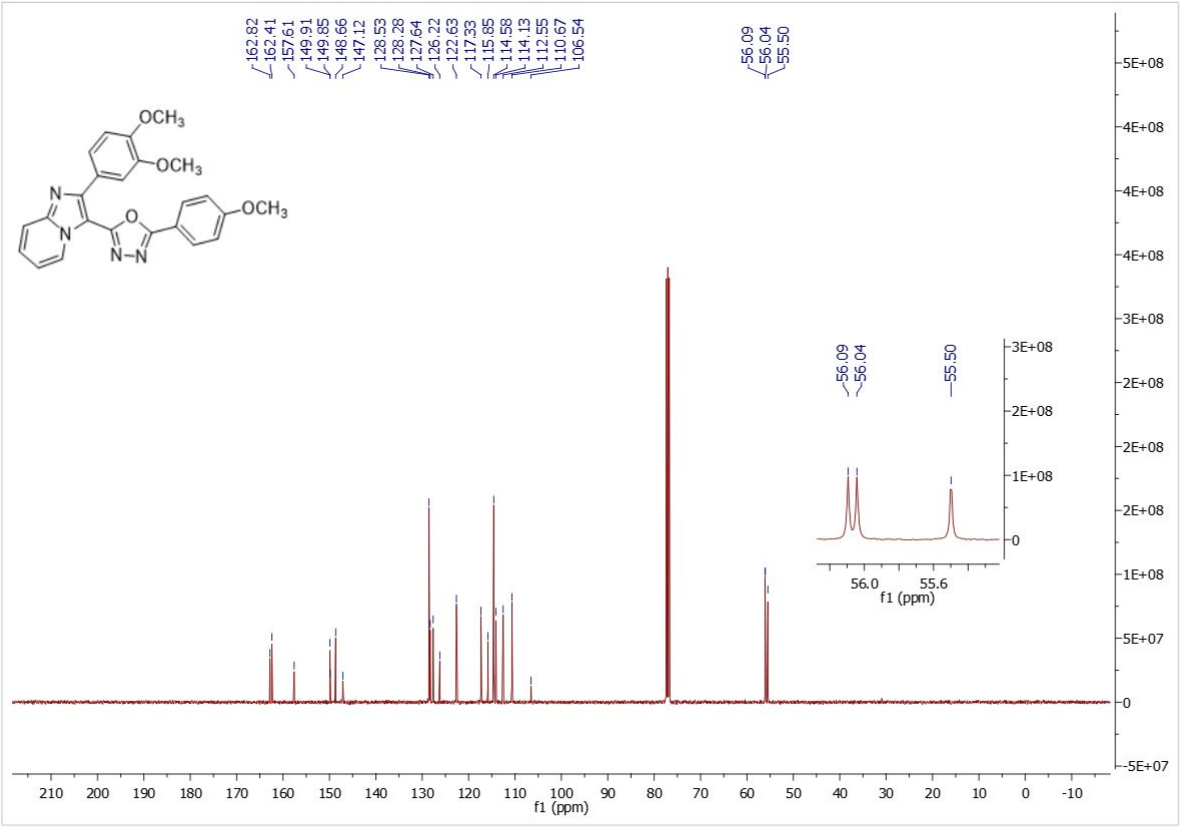

Figure S30. ^{13}C NMR spectrum of **iMPZ-15**

3. HRMS spectrum of the compounds

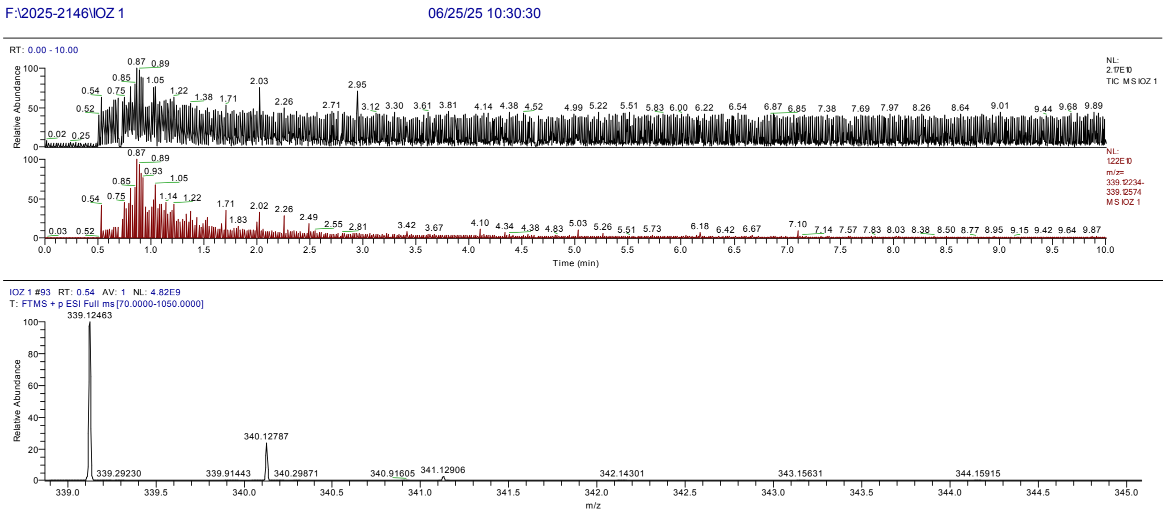

Figure S31. HRMS spectrum of compound **iMPZ-1**

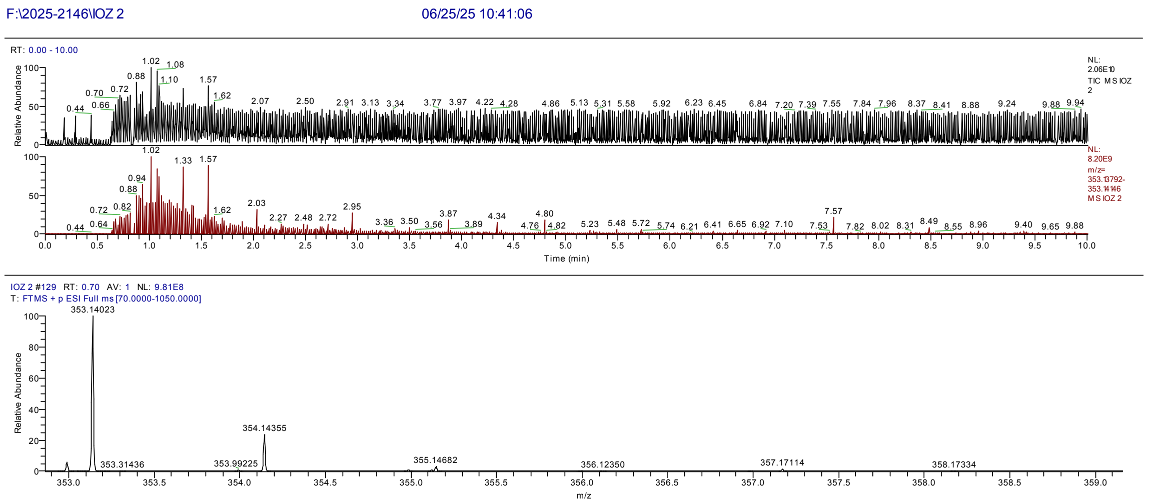

Figure S32. HRMS spectrum of compound **iMPZ-2**

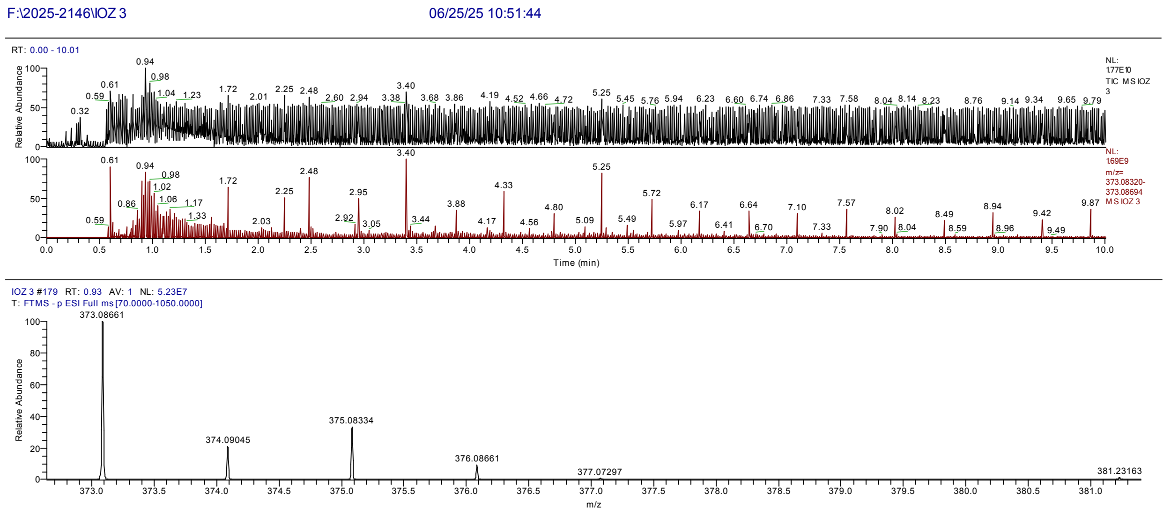

Figure S33. HRMS spectrum of compound **iMPZ-3**

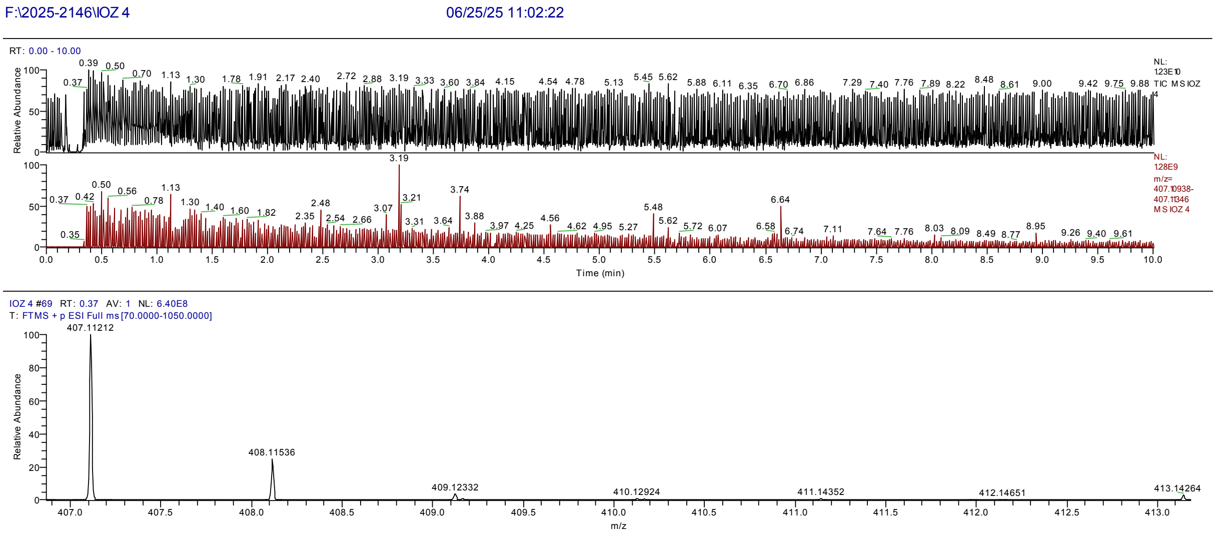

Figure S34. HRMS spectrum of compound **iMPZ-4**

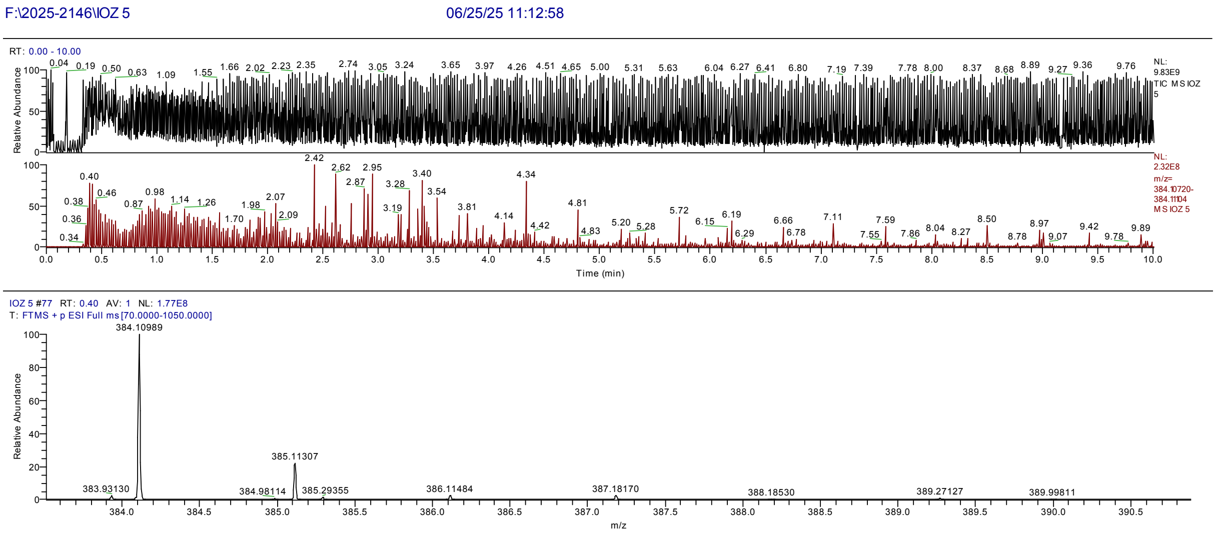

Figure S35. HRMS spectrum of compound **iMPZ-5**

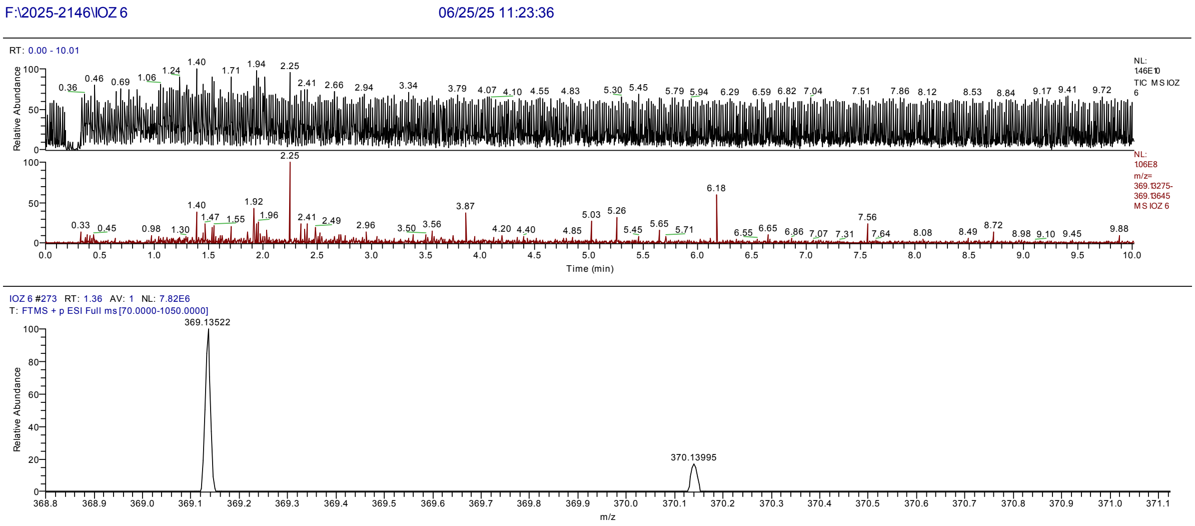

Figure S36. HRMS spectrum of compound **iMPZ-6**

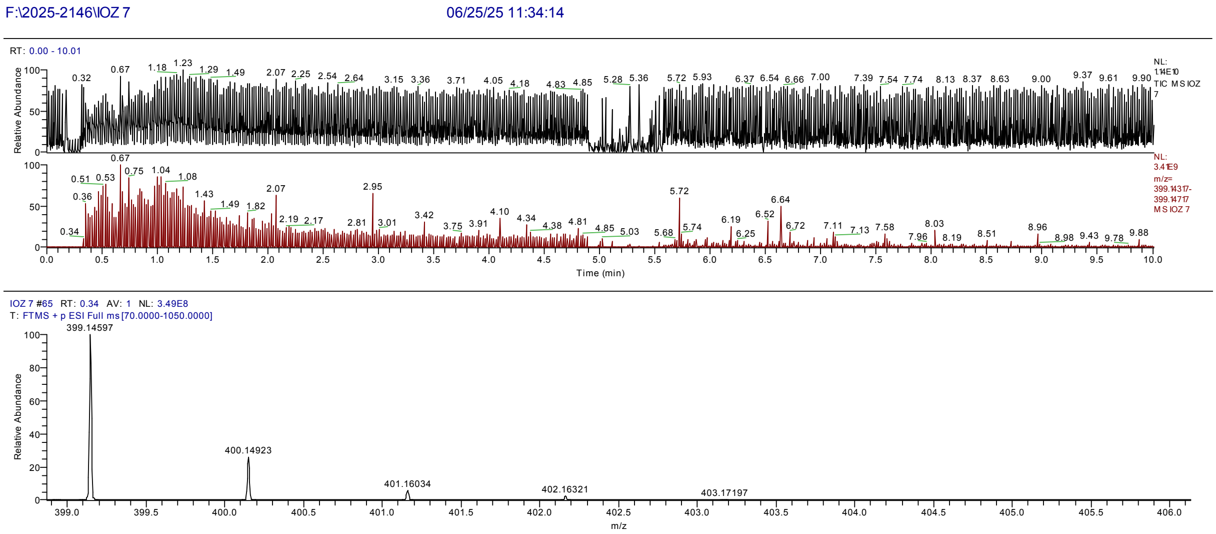

Figure S37. HRMS spectrum of compound **iMPZ-7**

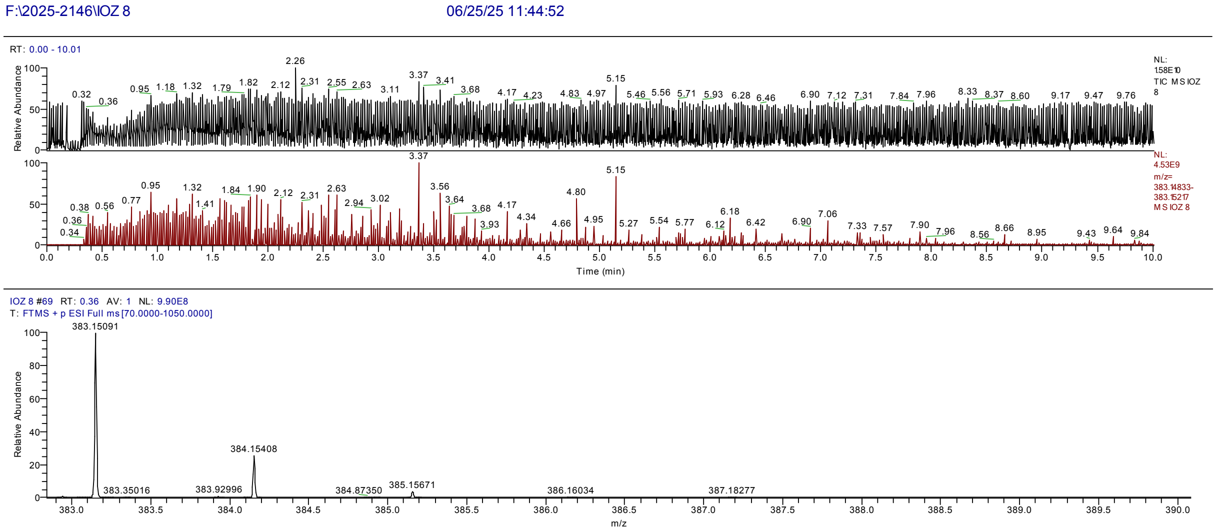

Figure S38. HRMS spectrum of compound **iMPZ-8**

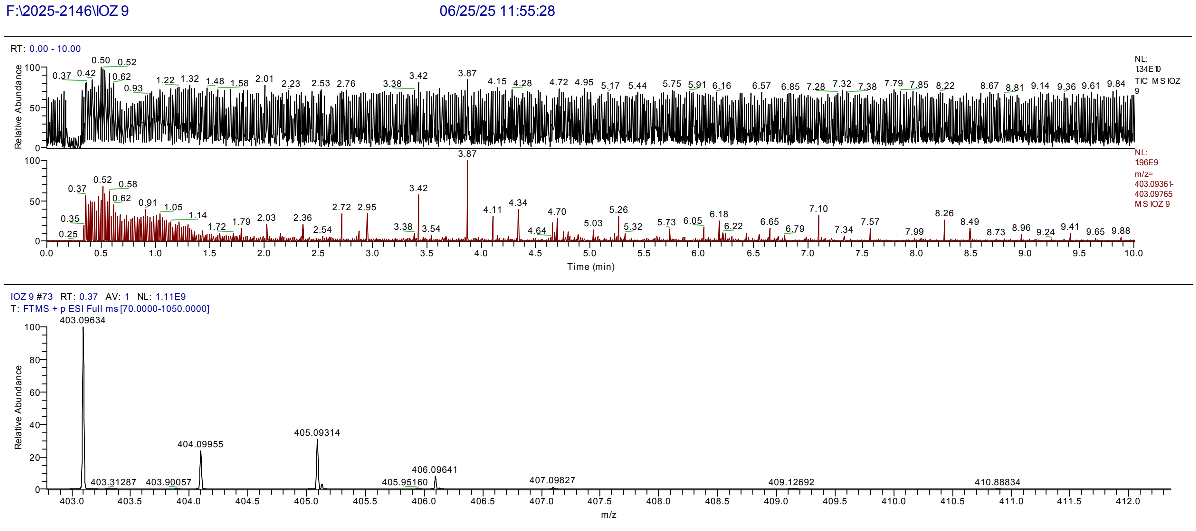

Figure S39. HRMS spectrum of compound **iMPZ-9**

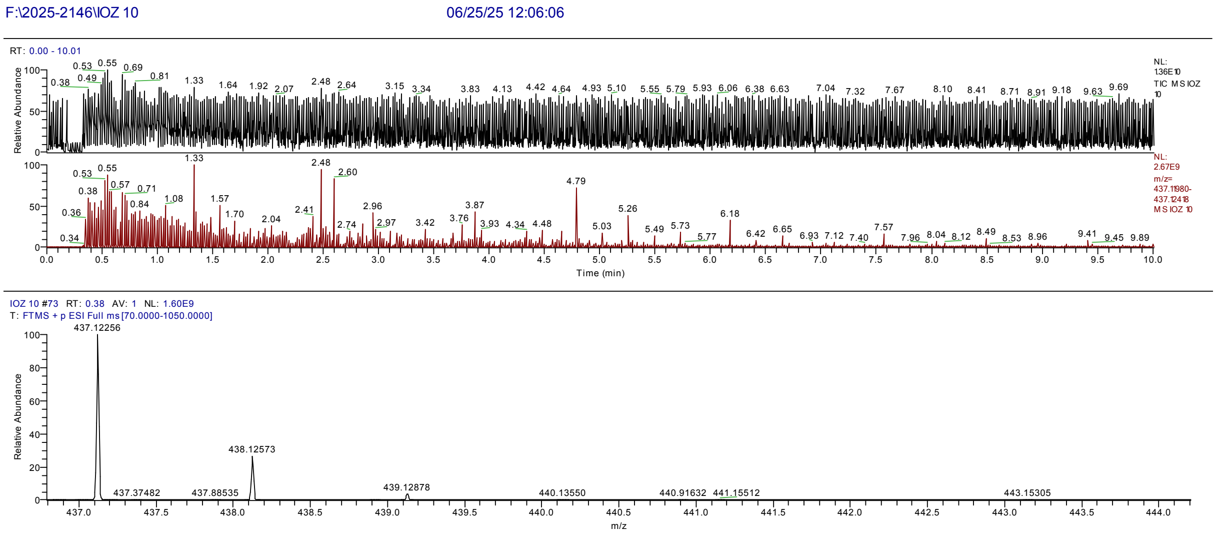

Figure S40. HRMS spectrum of compound **iMPZ-10**

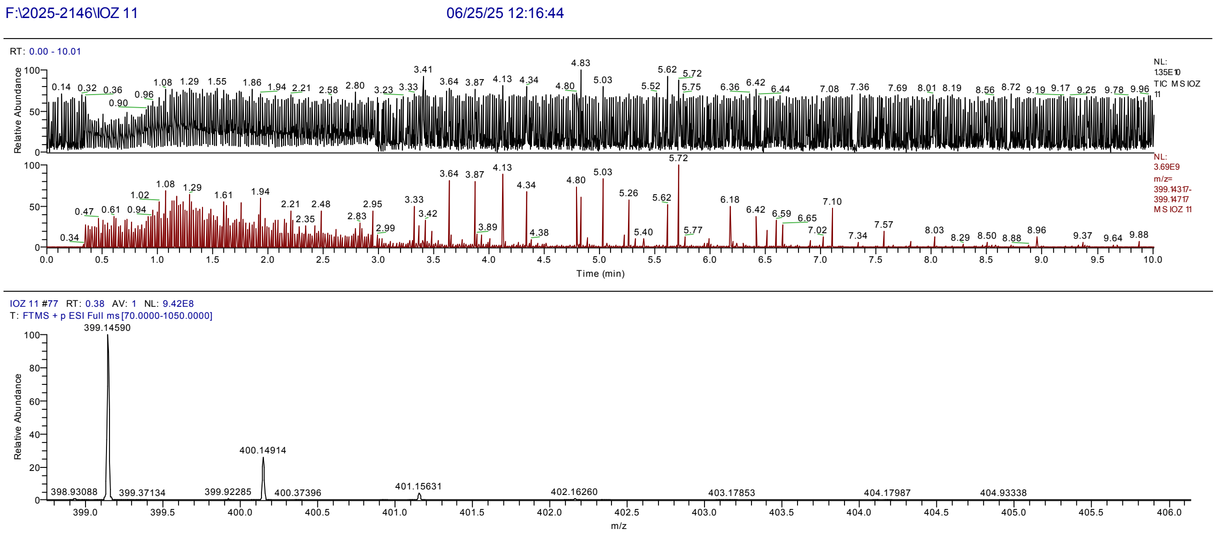

Figure S41. HRMS spectrum of compound **iMPZ-11**

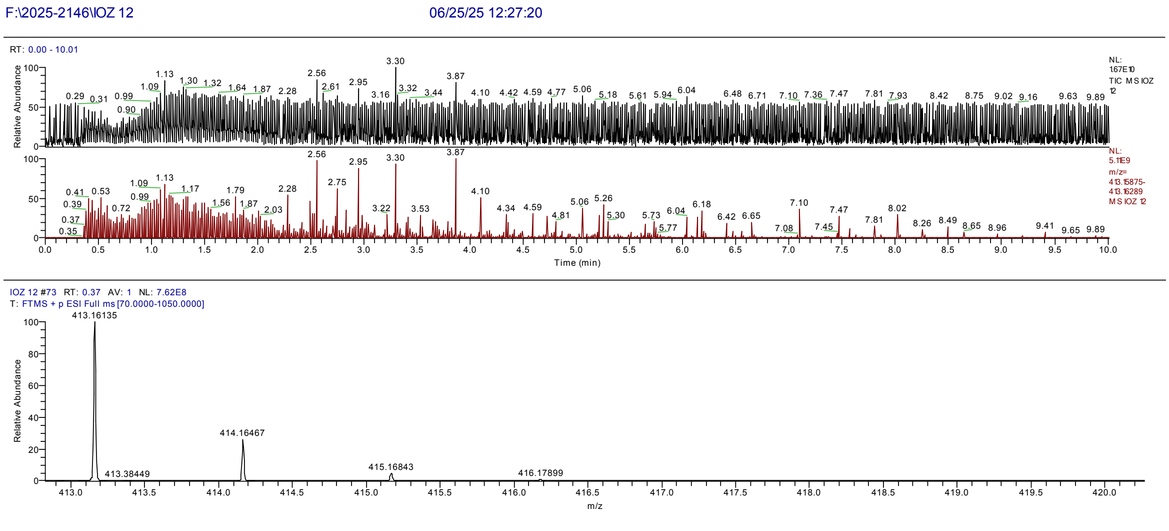

Figure S42. HRMS spectrum of compound **iMPZ-12**

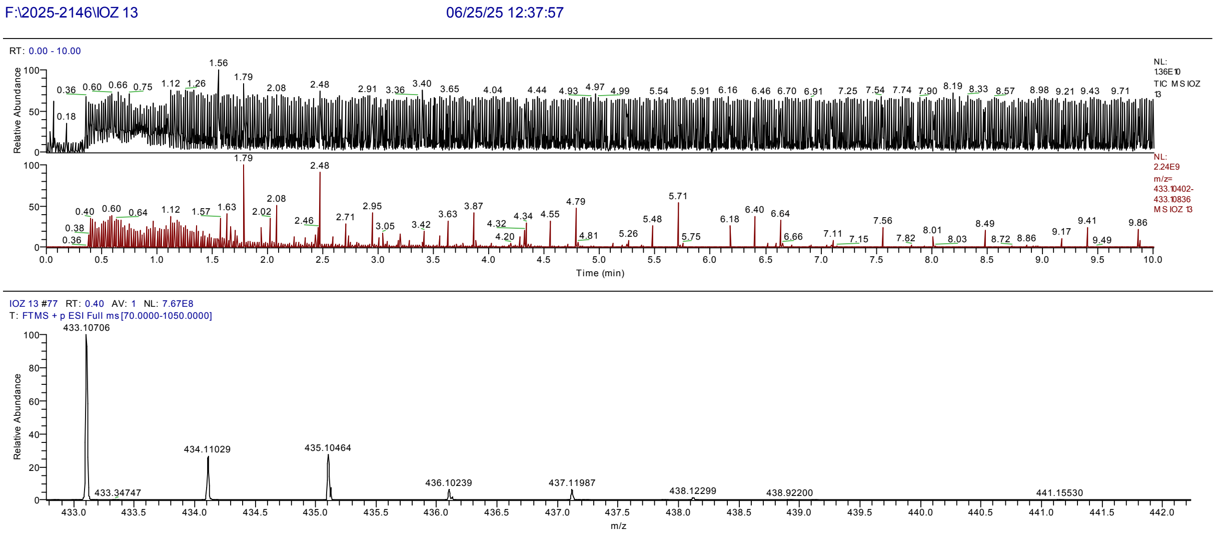

Figure S43. HRMS spectrum of compound **iMPZ-13**

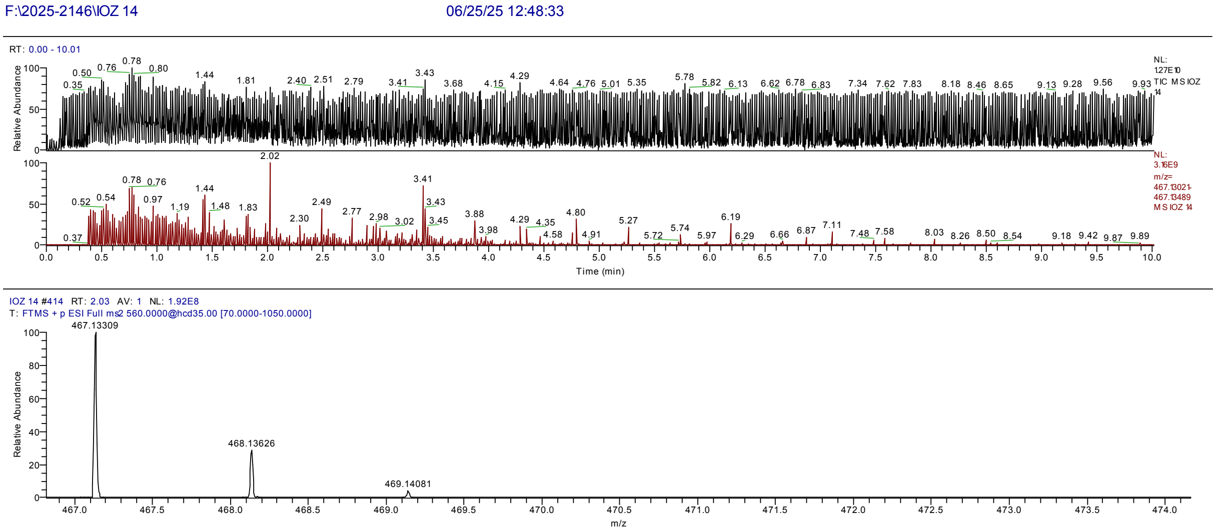

Figure S44. HRMS spectrum of compound **iMPZ-14**

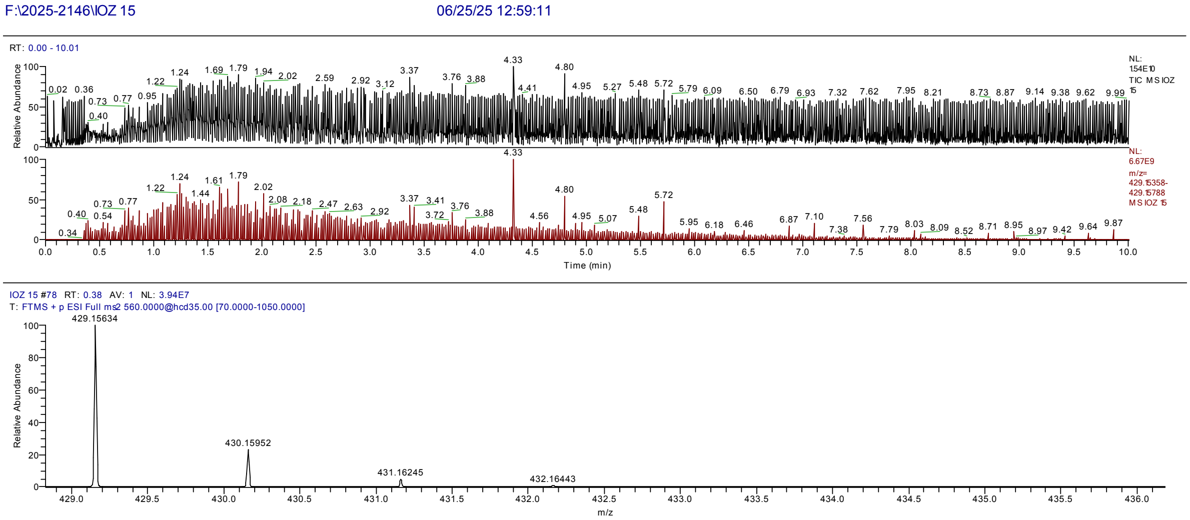

Figure S45. HRMS spectrum of compound **iMPZ-15**
